# Investigating the relationship between ATP synthase and the TCA cycle by crosslinking mass spectrometry

**DOI:** 10.1038/s41467-026-74730-5

**Published:** 2026-06-23

**Authors:** Laura Pérez Pañeda, Jelena Misic, Tereza Kadavá, Nils-Göran Larsson, Albert J. R. Heck

**Affiliations:** 1 Biomolecular Mass Spectrometry and Proteomics, Bijvoet Center for Biomolecular Research and Utrecht Institute for Pharmaceutical Sciences, University of Utrecht, Utrecht, The Netherlands; 2 Department of Medical Biochemistry and Biophysics, Karolinska Institutet, Stockholm, Sweden

**Keywords:** Mitochondrial proteins, Proteomic analysis

## Abstract

Mitochondrial oxidative phosphorylation (OXPHOS) comprises multi-subunit protein complexes that operate in coordination with the tricarboxylic acid (TCA) cycle to generate ATP. Although these systems are metabolically interconnected, complex II is generally regarded as the only direct structural link between OXPHOS and TCA cycle. Here, we combine in-solution crosslinking mass-spectrometry (XL-MS), quantitative proteomics, complexome profiling and blue native PAGE (BN-PAGE) to explore how ATP synthase (complex V) is positioned within the mitochondrial metabolic network under physiological and pathological conditions. We demonstrate that in murine wild-type hearts, the F₁ catalytic head of ATP synthase forms extensive contacts with TCA cycle enzymes, establishing a previously unanticipated spatial link between OXPHOS and central carbon metabolism. We further report that loss of the mitochondrial RNA-stabilizing protein LRPPRC, which disrupts mtDNA gene expression in the mouse heart, results in ATP synthase destabilization and enhanced F_1_-TCA cycle interactions. Moreover, ATP synthase dysfunction promotes binding of the ATPase inhibitory factor 1 (ATIF1) to the F₁ head via its N-terminal inhibitory region, shifting the ATP synthase toward an energy-preserving state. Together, our findings show that impaired mitochondrial gene expression leads to secondary ATP synthase remodeling and reshaping of its interaction landscape, revealing how mitochondria may adapt to bioenergetic stress.

## Abstract (web summary)

Mitochondria convert nutrients into ATP through coordinated molecular machines and metabolic pathways. Here, the authors show that ATP synthase contacts TCA cycle enzymes in mouse heart and that defective mitochondrial gene expression reshapes this interaction network.

## Introduction

Mitochondria are essential organelles present in nearly all eukaryotic cells with essential functions in energy conversion, metabolism, and signaling. Their main bioenergetic function is carried out by the oxidative phosphorylation (OXPHOS) system, which generates the majority of cellular ATP. OXPHOS comprises the respiratory chain complexes (I–IV) and ATP synthase (complex V), all embedded in the inner mitochondrial membrane. In mammals, it is well established that respiratory chain complexes can assemble into higher-order supramolecular structures known as supercomplexes^1–4^. However, the importance of these assemblies for mitochondrial bioenergetics has been challenged^5–7^ and their in vivo role remains to be fully elucidated. Furthermore, ATP synthase forms higher-order oligomers that have a well-defined architectural role in shaping the inner mitochondrial membrane folding^8,9^. The ATP synthase monomers associate to form dimers^3^, which further arrange into rows along the tips of cristae membranes to induce the typical curvature.

In addition to its architectural role, ATP synthase serves as a central energy-conversion machinery in mitochondria. It uses the proton-motive force generated by the respiratory chain (complexes I, III, and IV) to synthesize ATP through a rotary catalytic mechanism^10–12^. Each monomer is composed of two major domains, the soluble F₁ catalytic head and the membrane-embedded F_O_ proton channel, which are connected by the peripheral stalk^13,14^. When the proton-motive force collapses, for example, during transient mitochondrial depolarization, limited substrate or oxygen availability, ischemia-like conditions or respiratory chain dysfunction, ATP synthase can reverse its direction to hydrolyze ATP to help maintain the inner membrane potential^15^. This reverse activity is tightly regulated by the endogenous inhibitory factor ATIF1, which is a small protein (~10 kDa) that binds the F₁ head in a pH-dependent manner and prevents wasteful ATP hydrolysis^16,17^. In addition to its canonical role in inhibiting reverse ATP synthase activity, ATIF1 has also been implicated in mitochondrial cristae organization, as well as in mitophagy, Ca^2+^ handling, metabolic rewiring and ROS-dependent cell-survival signaling in specific cellular or disease contexts^18–23^.

The function of ATP synthase relies on subunits and assembly factors encoded by two genetic systems, namely the nuclear genome and mtDNA. Because of this dual genetic origin, the synthesis and import of nuclear-encoded components must be coordinated with the expression of mtDNA-encoded subunits to ensure accurate assembly and maturation of the complex. Mutations in ATP synthase subunits, defects affecting mtDNA maintenance or gene expression and mutations in assembly or regulatory factors can all compromise the integrity of the ATP synthase complex, leading to the accumulation of incomplete intermediates^24–28^. These molecular defects ultimately impair ATP synthase activity, cause mitochondrial dysfunction and contribute to the pathogenesis of numerous human disorders, including cardiovascular, neurodegenerative and neurodevelopmental diseases^29^.

ATP synthase operates in tight bioenergetic cooperation with both the respiratory chain complexes and the tricarboxylic acid (TCA) cycle, which provides reducing equivalents to sustain electron flow and proton-motive force generation. Complex II (succinate dehydrogenase) is unique in this regard, as it functions simultaneously as a TCA cycle enzyme and as an integral component of the OXPHOS system, thereby forming a direct structural and functional bridge between the two pathways. Electrons from NADH, produced by multiple oxidative steps of the TCA cycle, are transferred to the respiratory chain through complex I, whereas those from succinate oxidation enter via complex II. This metabolic coupling is essential for efficient energy conversion; however, it has remained elusive whether additional physical connections, besides those involving complex II, exist between the TCA cycle enzymes and the OXPHOS system.

For long, it has been hypothesized that metabolic enzymes, including those of the TCA cycle, can associate to form metabolons to facilitate substrate channeling and coordinate metabolic flux through interconnected pathways^30–32^. However, such interactions are typically transient, dynamic and weak, preventing their detection by most conventional biochemical or structural approaches. To capture these interactions and determine their spatial organization, advanced structural proteomics approaches are required. In-solution crosslinking mass spectrometry (XL-MS) has recently emerged as a powerful tool to investigate protein organization in intact mitochondria^33–38^. By covalently linking proximal amino acid residues, this technique enables direct mapping of protein-protein interactions arising from close spatial proximity. While most biochemical and structural methods require detergents and partial mitochondrial solubilization, XL-MS preserves the native environment in mitochondria, capturing both stable and transient interactions at a proteome-wide scale^39,40^. This approach therefore provides a unique opportunity to uncover functional protein networks in situ, within their physiological context.

Here, we apply in-solution XL-MS to investigate the organization of the OXPHOS interaction network in the mouse heart under different conditions. Although extensive structural information exists for individual OXPHOS complexes and their higher-order assemblies^34,41,42^, much less is known about how these complexes interact with other metabolic pathways within mitochondria. We perform XL-MS on intact heart mitochondria to map protein-protein interactions under normal physiological conditions and in a tissue-specific *Lrpprc* knockout mouse model with impaired mtDNA gene expression in the heart. Our XL-MS analyses reveal previously unrecognized associations between the F_1_ domain of ATP synthase and multiple TCA cycle enzymes in wild-type hearts. These interactions are further enhanced under mitochondrial dysfunction, causing compromised ATP synthase integrity in the *Lrpprc* knockout hearts. In addition to the TCA cycle enzymes, several other metabolic enzymes also show increased association with the F₁ head in the absence of LRPPRC. Notably, in vivo binding of the N-terminal inhibitory domain of ATIF1 to the F₁ head of ATP synthase is observed in *Lrpprc* knockout hearts. Our findings provide a link between the structural remodeling of ATP synthase and the inhibitory activation of ATIF1 under mitochondrial dysfunction caused by LRPPRC deletion.

## Results

### Mitochondrial ATP synthase associates with the TCA cycle enzymes in the mouse heart

To gain a broader understanding of the spatial organization of OXPHOS in mitochondria, we first revisited published in-solution XL-MS data from intact murine heart mitochondria^5^. While the original analyses primarily focused on protein–protein interactions within and between the different respiratory chain complexes in heart mitochondria with normal and deficient formation of respirasomes (supercomplex I–III_2_–IV), the underlying dataset provided an opportunity to explore additional mitochondrial protein associations.

**Fig. 1:**
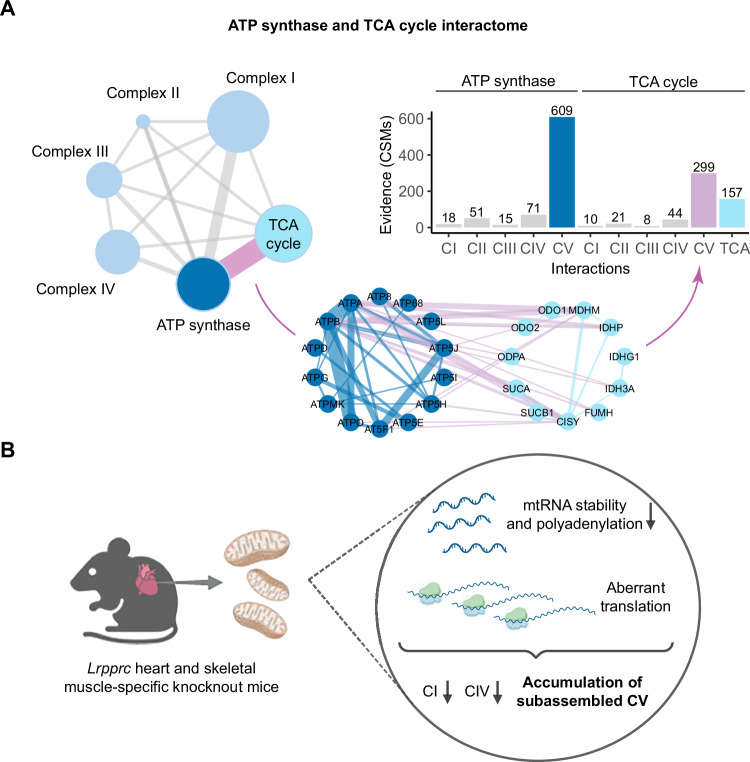
ATP synthase (complex V) interacts strongly with TCA cycle enzymes in the mouse heart. **A** Network plot illustrating the interactions between the OXPHOS complexes and the TCA cycle enzymes. Specific focus is given to the ATP synthase and TCA cycle interactions. Data represents the sum of *n* = 3 replicates from wild-type heart mitochondria, reanalyzed data^5^. Thickness of the lines relates to the number of cross-links observed. The interaction evidence was defined by the crosslink spectral matches (CSMs) shown as a histogram. **B** Schematic representation of the molecular phenotype found in the hearts of heart and skeletal muscle-specific *Lrpprc* knockout mice^24,43–45^, abbreviations: complex I (CI), complex IV (CIV), ATP synthase (CV). Created in BioRender. Misic, J. (2026) https://BioRender.com/dphdap1.

Here, we focused especially on identified cross-links between OXPHOS complexes and central metabolic pathways within mitochondria. The wild-type mouse heart mitochondria dataset revealed extensive interactions between the OXPHOS complexes and enzymes of the TCA cycle (Fig. 1A and Supplementary Fig. 1). Although complex II has been considered the primary structural bridge between these two systems, the ATP synthase exhibited the strongest connectivity to TCA cycle enzymes among all OXPHOS complexes (Fig. 1A and Supplementary Fig. 1). Crosslinks were detected across both the soluble F_1_ and membrane-embedded F_O_ subunits of ATP synthase, and involved multiple enzymes of the TCA cycle, including most notably citrate synthase (CISY), isocitrate dehydrogenase isoforms (IDHP, IDH3A), 2-oxoglutarate dehydrogenase subunits (ODPA, ODO1, and ODO2), succinyl-CoA ligase subunits (SUCA, SUCB1), fumarate hydratase (FUMH) and malate dehydrogenase (MDHM) (Fig. 1A and Supplementary Fig. 1). Most detected crosslinks between TCA cycle enzymes and ATP synthase were formed with subunits of the F_1_ catalytic head, primarily the α (ATPA) and β (ATPB) subunits (Fig. 1A and Supplementary Fig. 1). Interestingly, the number of such crosslinks was roughly twofold lower than those within the ATP synthase itself, yet about twice as abundant as the crosslinks detected between individual TCA cycle enzymes (Supplementary Fig. 1). Collectively, these data suggest that OXPHOS and the TCA cycle are structurally closely coupled in cardiac mitochondria, with ATP synthase likely positioned at the interface between these two systems.

### Knockout of *Lrpprc* in the mouse heart perturbs the integrity of the F_O_ portion of ATP synthase

Next, we proceeded to investigate how the identified ATP synthase—TCA cycle interactions observed in the published dataset would be affected in mitochondrial dysfunction by performing experiments on heart mitochondria from heart- and skeletal-muscle-specific *Lrppr*c knockout mice, a well-established model of impaired mitochondrial gene expression^24,43–45^. LRPPRC is an RNA-binding protein required for polyadenylation, stabilization, and coordinated translation of mitochondrial mRNAs (mtRNAs) (Fig. 1B). In the heart, loss of LRPPRC impairs the synthesis of mtDNA-encoded OXPHOS subunits, leading to ATP synthase deficiency with an accumulation of F_1_ subassemblies and a major bioenergetic defect primarily attributed to ATP synthase dysfunction^24,43,45^. The *Lrpprc* knockout mice thus provide a robust model to investigate how ATP synthase deficiency and instability reshape its protein-interaction network.

**Fig. 2:**
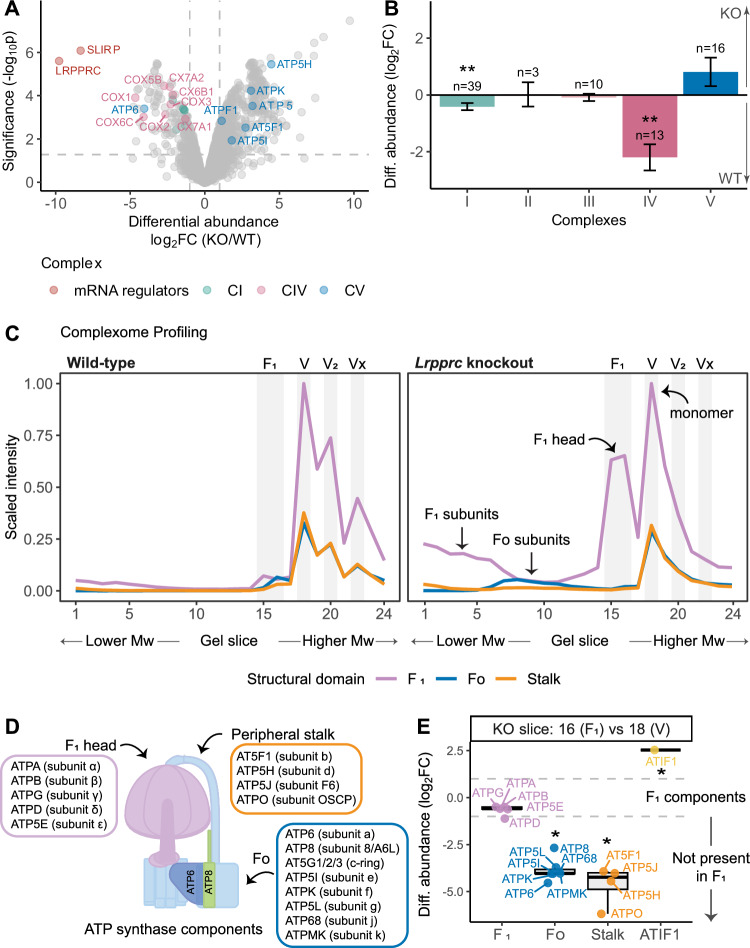
Bottom-up proteomics and complexome profiling analyses of heart mitochondria revealed destabilization of ATP synthase in the *Lrpprc* knockout heart mitochondria. **A** Differential abundance analysis of proteins detected in *Lrpprc* knockout (KO) versus wild-type (WT) heart mitochondria. The y-axis represents the -log_10_ *p*-value and the x-axis the log_2_ fold change (FC). Proteins related to mRNA regulation, complex I (CI), complex IV (CIV), and ATP synthase (CV) are highlighted with FDR < 5% and |log_2_FC| > 1. FDR was calculated by a “moderated t-statistic” two-sided test with Benjamini–Hochberg correction. Biological replicates, *n* = 3. **B** Bar plot representing the mean differential abundance of OXPHOS complex protein subunits calculated as log_2_ fold change. A positive fold change indicates higher abundance in the *Lrpprc* knockout compared to wild-type heart mitochondria. *n* represents the number of subunits identified per complex. Adjusted *p*-values were calculated by a two-sided Student’s *t* test with Benjamini–Hochberg correction: CI 0.006; CII 0.966; CIII 0.571; CIV 0.002; and CV 0.231. Biological replicates, *n* = 3; error bars indicate mean ± SEM. **C** Migration profiles of ATP synthase assemblies in wild-type and *Lrpprc* knockout heart mitochondria. The y-axis represents the structural-domain intensity normalized to the maximum per lane; the x-axis represents the 24 gel slices ordered by increasing molecular weight. The profiles indicate the presence of monomers (V), dimers (V_2_) and oligomers (V_x_) in the wild-type. *Lrpprc* knockout mitochondria show reduced dimeric and oligomeric structures and the appearance of an ATP synthase subassembly corresponding to the F_1_ head (F_1_, ~325–390 kDa). F_1_ masses were estimated using the migration and masses of known complexes (Supplementary Fig. 11). Median of *n* = 3 biological replicates. **D** Illustration of ATP synthase components grouped by F_1_ head, Fo and peripheral stalk. **E** Box plot of the differential abundance analysis of complexome profiling slices 16 (F_1_) and 18 (V_1_), corresponding to the F_1_ assembly and ATP synthase monomer, respectively. The y-axis represents the log2 fold change; the x-axes the ATP synthase structural components and ATIF1. Boxplots indicate median (middle line), 25th and 75th percentile (box); maximum and minimum values (whiskers), without values further than 1.5 times the inter-quartile range. FDR for each protein was calculated as in (**A**). Biological replicates, *n* = 3. *Adjusted *p*-value < 0.01, **adjusted *p*-value < 0.05. Source data are provided as a Source data file.

We first performed label-free quantitative (bottom-up) proteomics on isolated heart mitochondria from wild-type and tissue-specific *Lrpprc* knockout mice to define global relative changes in mitochondrial protein abundance. The proteomics analyses revealed an expected profound downregulation of LRPPRC in the knockouts (Fig. 2A). This was accompanied by a concomitant decrease in SLIRP, highlighting the essential role of LRPPRC in maintaining SLIRP stability, as we previously have reported^43–46^. Interestingly, we also observed reduced levels of several mtDNA-encoded OXPHOS subunits, including COX1, COX2, and COX3 (complex IV) and ATP6 (ATP synthase) (Fig. 2A). Notably, the abundance of ATP8, another mtDNA-encoded subunit of ATP synthase that, together with ATP6, forms part of the membrane-embedded F_O_ domain, was found to be unchanged. In contrast, several nuclear-encoded ATP synthase subunits localized either to the F_O_ domain (ATPK, ATP5L, and ATP5I) or the peripheral stalk (ATP5H, AT5F1) were upregulated, while the abundance of the catalytic F_1_ subunits remained unaffected in the LRPPRC knockout hearts (Fig. 2A). These changes of levels of ATP synthase subunits likely represent a compensatory response to its instability, reflected by the increased expression of assembly and biogenesis factors (Supplementary Data 1), including the ATP synthase F_1_ complex assembly factor 1 (ATPF1)^22^.

Analyses of global OXPHOS protein levels, in which the mean fold change of all identified subunits within each complex was compared between conditions, revealed a general decrease only in complexes I and IV (Fig. 2B) in the *Lrpprc* knockout hearts, in agreement with earlier reports^24,44^. Beyond the OXPHOS system, proteins typically associated with mitochondrial dysfunction, including those involved in apoptosis, protein degradation, stress responses and one-carbon metabolism, were also upregulated, consistent with previous findings by Kühl et al.^44^ (Supplementary Data 1). Moreover, we identified additional dysregulated proteins in the *Lrpprc* knockout heart mitochondria, such as components of the mitochondrial calcium uptake (MICU) complex, several solute carriers (SLCs) and tRNA aminoacylation-related enzymes (Supplementary Fig. 2).

We next performed proteomics-based complexome profiling to quantitatively map ATP synthase assemblies in wild-type and LRPPRC knockout heart mitochondria. Mitochondria were solubilized under mild detergent conditions and separated by Blue Native PAGE (BN-PAGE), after which gel lanes were excised into 24 slices and each slice was analyzed by LC-MS/MS. In wild-type mitochondria, ATP synthase subunits from all three structural domains (F_1,_ F_O_, and peripheral stalk, Fig. 2C, D) predominantly co-migrated in high-molecular-weight slices corresponding to monomeric (V), dimeric (V_2_) and oligomeric (V_x_) forms of ATP synthase. Loss of LRPPRC in the heart caused a pronounced redistribution of ATP synthase subunits across the BN-PAGE lanes. Peaks corresponding to intact ATP synthase dimers and oligomers were diminished, whereas the monomeric complex was relatively preserved. In parallel, F_1_ subunits accumulated in two discrete regions of the lane that lacked detectable F_O_ and peripheral stalk components. There was also a distinct lower-molecular-weight peak for F_O_ subunits, indicating destabilization and fragmentation of intact ATP synthase assemblies (Fig. 2C, E).

### Increased interactions between the partially released F_1_ domain of ATP synthase and metabolic enzymes in the *Lrpprc* knockout heart mitochondria

Given the ATP synthase deficiency and structural instability in *Lrpprc* knockouts^24,43^, we next examined how these alterations affect the interaction landscape of ATP synthase. To this end, we performed in-solution XL-MS analyses on intact mitochondria isolated from wild-type and *Lrpprc* knockout hearts using an optimal concentration of 0.5 mM disuccinimidyl sulfoxide (DSSO, Supplementary Fig. 3). The crosslinking data showed high reproducibility across biological replicates (Supplementary Fig. 4). To compare the two conditions, crosslinks were quantified based on MS1 intensities and differential abundance analyses of the interactions were performed (Supplementary Data 2). In brief, fold changes were calculated as the difference in total MS1 crosslink intensity of the interactions detected in the wild-type and *Lrpprc* knockout heart mitochondria. To ensure that the observed interaction changes were not confounded by protein abundance differences, we used the generated quantitative proteomics data as a reference for the evaluation of the interactomes.

**Fig. 3:**
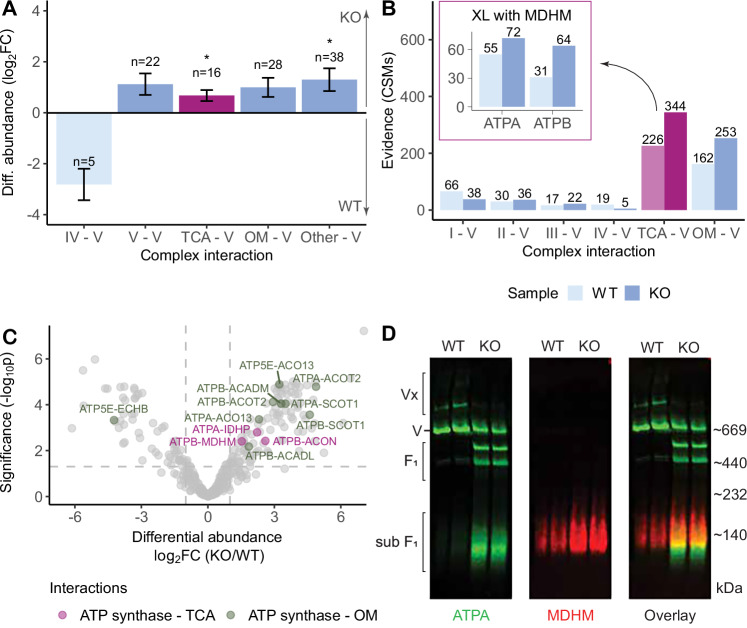
Crosslinking analyses of wild-type and *Lrpprc* knockout heart mitochondria revealed increased interactions between the F_1_ head of ATP synthase and metabolic enzymes. **A** XL-MS data. Bar plot representing the mean log_2_ fold change of the differential ATP synthase protein interactions with other OXPHOS complexes, TCA cycle proteins (TCA), other mitochondrial metabolic enzymes (OM) and the remaining mitochondrial matrix proteins (other). The fold change represents the difference in crosslink intensity between *Lrpprc* knockout and wild-type heart mitochondria. *n* is the number of protein interactions identified per group. The adjusted *p*-values were calculated by a two-sided Student’s *t* test with Benjamini–Hochberg correction: CIV-CV, 0.056; CV-CV, 0.059; TCA-CV, 0.047; OM-CV, 0.059; and Other-CV, 0.047. *Adjusted *p*-value < 0.01. Biological replicates, *n* = 3; error bars indicate mean ± SEM. **B** Evidence of protein-protein interactions calculated as crosslink spectral matches (CSMs) detected between ATP synthase and OXPHOS complexes and metabolic proteins. Wild-type and *Lrpprc* knockout heart mitochondria are represented in light and dark blue, respectively. ATP synthase-TCA cycle interactions are highlighted in pink. A detailed view of interactions between MDHM and ATP synthase proteins, ATPA and ATPB, is provided. Cumulative across *n* = 3 biological replicates. **C** Differential abundance analysis of the interactions detected in the *Lrpprc* knockout versus the wild-type heart mitochondria. The y-axis represents the -log_10_ *p*-value and the x-axis the log_2_ fold change, calculated as the difference in crosslink intensity between *Lrpprc* knockout and wild-type heart mitochondria. ATP synthase interactions with TCA cycle and other metabolic enzymes with FDR < 5% and |log_2_FC| > 1 are highlighted. FDR was calculated by a “moderated t-statistic” two-sided test with Benjamini–Hochberg correction. Biological replicates, *n* = 3. **D** Fluorescent Western blot of BN-PAGE-separated wild-type and *Lrpprc* knockout heart mitochondria after digitonin solubilization, co-incubated with antibodies against ATPA and MDHM and imaged in separate fluorescence channels, with the merged signal shown. The approximate positions of ATP synthase oligomers (V_x_), monomers (V), F_1_ assemblies (F_1_) and F_1_ subassemblies (sub F_1_) are indicated on the left side. *Lrpprc* knockout mitochondria show partial disassembly of ATP synthase with the subassembled F_1_ species comigrating with MDHM, among other proteins. Each lane represents one biological replicate. Representative immunoblot from three independent experiments with similar results. Source data are provided as a Source data file.

We first assessed the global interaction changes among the five OXPHOS complexes, metabolic enzymes, and other proteins present in the mitochondrial matrix in the *Lrpprc* knockouts. Differential changes were detected in crosslink intensity between ATP synthase and complex IV, between ATP synthase and TCA cycle enzymes and between ATP synthase and other proteins, among which were the components of the electron transfer flavoprotein system (ETFA, ETFB, and ETFD), ATP synthase assembly factors and regulators of its activity (Fig. 3A and Supplementary Data 2). Interactions involving ATP synthase (subunits ATPB and ATPO) and complex IV (subunits COX6A1, COX7A2, and COX5B) were reduced in the *Lrpprc* knockout mitochondria (Fig. 3A), likely because of the overall decrease in complex IV abundance (Fig. 2A, B). In contrast, interactions between ATP synthase and TCA cycle enzymes were markedly increased in the *Lrpprc* knockouts, as indicated by an almost twofold increase in total crosslink intensity (Fig. 3A). This increase was further supported by the higher number of identified interactions which were represented by the crosslink spectral matches (CSMs), with 226 and 344 detected in wild-type and *Lrpprc* knockout mitochondria, respectively (Fig. 3A, B). Loss of LRPPRC in the heart specifically enhanced crosslinking between the F_1_ head of the ATP synthase (subunits ATPA, ATPB, and ATP5E) and enzymes of the TCA cycle (ACON, IDHP, MDHM) as well as the enzymes involved in fatty-acid β-oxidation (acyl-coenzyme A thioesterase 2, ACOT2; acyl-coenzyme A thioesterase 13, ACOT13; medium-chain specific acyl-CoA dehydrogenase, ACADM, and ACADL) and ketone-body metabolism (succinyl-CoA:3-ketoacid coenzyme A transferase 1, SCOT1) (Fig. 3C). Notably, both the F_1_ head subunits and all metabolic enzymes involved in these interactions displayed unchanged abundance in the proteomics and western blot data (Supplementary Figs. 5 and 6), showing that the elevated crosslink intensity arises from enhanced physical association between these proteins in the *Lrpprc* knockout hearts rather than changes in their expression levels.

To validate the increased interactions in the *Lrpprc* knockouts detected by XL-MS, we examined whether subunits of the F_1_ head and TCA cycle enzymes co-migrate on BN-PAGE under mild mitochondrial solubilization conditions. We focused on the F_1_ subunit ATPA and the TCA cycle enzyme MDHM, as they exhibited one of the strongest increases in crosslink intensity among TCA cycle enzymes between the two mouse systems (Fig. 3D and Supplementary Fig. 7A). We confirmed that loss of LRPPRC led to a marked reduction in the dimeric and oligomeric ATP synthase population and the accumulation of three distinct F_1_ subassemblies containing ATPA (Fig. 3D), consistent with complexome profiling data (Fig. 2C). Notably, the lower-molecular-weight F_1_ species co-migrated with MDHM, in agreement with the association detected by XL-MS (Fig. 3A–C). Moreover, the MDHM signal on BN-PAGE appeared to be more intense and diffuse in *Lrpprc* knockout mitochondria, despite unchanged total MDHM protein levels (Supplementary Figs. 5 and 6).

**Fig. 4:**
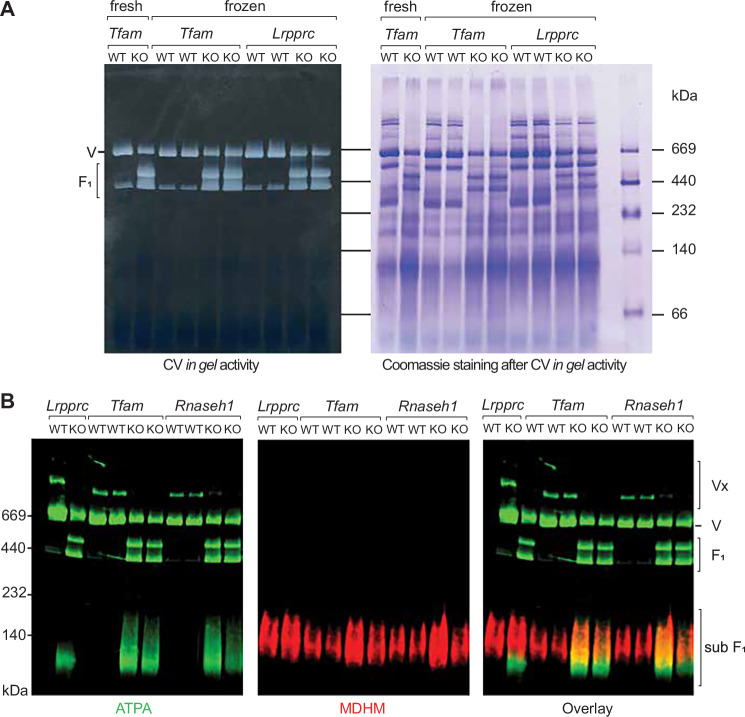
Co-migration of MDHM and ATPA in mouse models with disrupted mtDNA maintenance and expression. **A** BN-PAGE analyses of heart mitochondria followed by ATPase (CV) in gel activity show an increase in ATP synthase subassemblies in *Lrpprc* and *Tfam* knockouts (KO). No difference is observed between freshly isolated and frozen mitochondria samples after digitonin solubilization. **B** Fluorescent western blot of BN-PAGE-separated heart mitochondria from tissue-specific *Lrpprc*, *Tfam,* and *Rnaseh1* knockouts (KO) and their corresponding controls (WT), probed with antibodies against ATPA and MDHM and overlay of both signals. The position of the ATP synthase oligomers (V_x_), monomers (V), F_1_ head assemblies (F_1_) and F_1_ head subassemblies (sub F_1_) is indicated (**A**, **B**). Each lane represents one biological replicate. Representative gels and immunoblots from three independent experiments with similar results. Source data are provided as a Source data file.

Following the BN-PAGE analyses, we also assessed ATPase in gel activity in the *Lrpprc* knockout mitochondria. The monomeric ATP synthase forms and the two high-molecular-weight F_1_ assemblies retained ATP hydrolytic activity, whereas the lower-molecular-weight F_1_ species showed no catalytic activity (Fig. 4A), indicating that the latter represent a partially disrupted F_1_ head. Together, these findings show that mitochondrial dysfunction caused by LRPPRC loss promotes the reorganization of MDHM into higher-molecular-weight assemblies, some of which may include partially assembled F_1_ species of ATP synthase.

Next, we asked whether the increased interaction between ATPA and MDHM is unique to LRPPRC deficiency or if it represents a more general consequence of impaired mtDNA maintenance and expression and subsequent ATP synthase dysfunction and instability. To this end, we used BN-PAGE to examine heart mitochondria of other knockout mouse models affecting distinct steps of mtDNA maintenance and gene expression (Fig. 4B and Supplementary Fig. 7B). The studied mutants included heart and skeletal muscle-specific knockouts of TFAM, a mtDNA packaging and transcription factor, and RNase H1, an enzyme required for the initiation and completion of mtDNA replication^47,48^. Similar to the *Lrpprc* knockout, lower-molecular-weight F_1_ species co-migrated with MDHM, which displayed a stronger and more diffuse signal in both *Tfam* and *Rnaseh1* knockout mitochondria (Fig. 4B). These findings suggest that the enhanced association between ATP synthase and MDHM, used here as a representative TCA cycle enzyme, likely reflects a broader response to ATP synthase dysfunction and instability, which arises secondary to altered mitochondrial gene expression.

### LRPPRC loss induces ATIF1-mediated regulatory remodeling at the catalytic F_1_ domain of ATP synthase

**Fig. 5:**
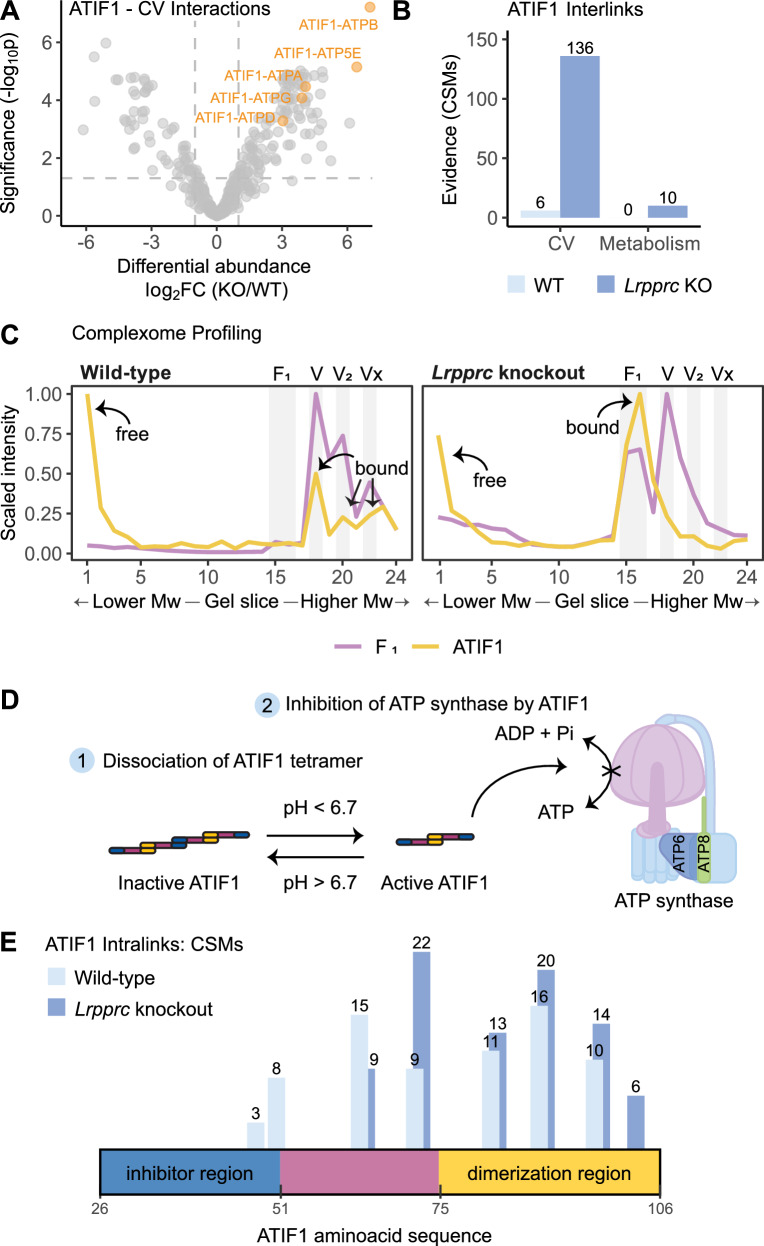
ATIF1 dissociates into dimers upon mitochondrial dysfunction to inhibit the ATP synthase. **A** Differential abundance analysis of the interactions between ATP synthase and ATIF1 as detected by XL-MS analyses of *Lrpprc* knockout (KO) and wild-type (WT) heart mitochondria. The y-axis represents the -log10 *p*-value and the x-axis the log_2_ fold change (FC). The fold change was calculated as the difference in crosslink intensity between the *Lrpprc* knockout and wild-type heart mitochondria. ATP synthase (CV) interactions with metabolic proteins with an FDR < 5% and |log_2_FC| > 1 are highlighted. FDR was calculated by a “moderated t-statistic” two-sided test followed by Benjamini–Hochberg correction. Number of biological replicates, *n* = 3. **B** Evidence of protein interactions represented by the crosslink spectral matches (CSMs) detected in the WT (light blue) and the *Lrpprc* knockout (dark blue) samples, showing interactions between ATIF1 and ATP synthase or proteins related to mitochondrial metabolism in the matrix. Cumulative across *n* = 3 biological replicates. **C** Migration profiles of ATIF1 with ATP synthase assemblies in wild-type and *Lrpprc* knockout heart mitochondria. The y-axis represents the intensity of each assembly normalized to the maximum intensity per lane, and the x-axis represents the 24 gel slices ordered from lower to higher molecular weight. The profiles indicate co-migration of ATIF1 with ATP synthase assemblies in the wild-type heart mitochondria, with a shift in co-migration of ATIF1 towards the F_1_ head and a reduction in free ATIF1 in the *Lrpprc* knockout heart mitochondria. The gel slices where the ATP synthase assemblies migrate are highlighted in grey: F_1_ head (F_1_), monomers (V), dimers (V_2_), oligomers (V_x_). Median of *n* = 3 biological replicates. **D** Schematic representation of the dissociation of the ATIF1 tetramer into dimers, followed by binding and inhibition of the ATP synthase. The F_1_ region of the ATP synthase is colored in pink. **E** Distribution of ATIF1 intralinks across the protein sequence calculated as crosslink spectral matches (CSMs) detected for the interaction between ATIF1 proteins in the wild-type (light blue) and the *Lrpprc* knockout (dark blue) samples. The ATIF1 regions were defined based on their functional properties: inhibition (residue 26–52), dimerization (residue 74–106), and other (residue 53–63). Cumulative across *n* = 3 biological replicates. Source data are provided as a Source data file.

We next examined interactions between ATP synthase subunits and suggested modulators of its activity. Among the dysregulated associations, we observed increased interlinks with PPIF (Cyclophilin D) and ATIF1 in the *Lrpprc* knockout mitochondria. Interactions between the ATPB subunit of the F_1_ head and PPIF were increased (Supplementary Fig. 8). The precise mechanism by which PPIF exerts its regulatory effect on ATP synthase remains unknown. Although an interaction between PPIF and OSCP (ATPO) has been reported biochemically^49^, we did not observe OSCP cross-links in our XL-MS dataset. However, we detected PPIF cross-links with the ATPA and ATPB subunits of ATP synthase. Importantly, PPIF–ATPB interactions were increased in LRPPRC knockout mitochondria, consistent with stress-induced remodeling of the F_1_ head and an enhanced susceptibility to permeability transition pore activation^50^, previously reported due to PPIF binding to peripheral stalk subunits^49^. ATIF1, in contrast, acts as a regulatory protein with a dual role in stabilizing ATP synthase dimers and modulating the catalytic activity of the F_1_ domain^51^. In the absence of LRPPRC, we observed a vast increase in the detected cross-links of ATIF1 to the F_1_ head of ATP synthase (Fig. 5A, B). These findings are consistent with cryo-EM data showing that ATIF1 exerts its inhibitory effect through direct binding to the F_1_ catalytic domain^21,52,53^. We further corroborated these observations by complexome profiling. In wild-type heart mitochondria, ATIF1 was present in its free form and in higher-molecular-weight fractions where it comigrated with intact ATP synthase monomers, dimers and oligomers (Fig. 5C). In contrast, in the absence of LRPPRC in the heart, the non-bound ATIF1 pool was markedly reduced, as were the fractions comigrating with ATP synthase oligomers. Instead, the peak wherein ATIF1 predominantly comigrates with F_1_ assemblies had intensified. Together, these data support that loss of LRPPRC destabilizes ATP synthase and promotes accumulation of detached F_1_ head, a condition under which ATIF1 would be required and recruited to restrain wasteful ATP hydrolysis by the F_1_ head.

Previous in vivo studies in cells and mice have shown that ATIF1 itself exists in inactive tetrameric or oligomeric forms, as well as in an active dimeric form that binds to ATP synthase^21,22,51,54^. To further investigate the structural basis of ATIF1 binding to ATP synthase in mitochondria under native conditions, we examined the conformational differences between its active and inactive forms. ATIF1 was shown to undergo pH-dependent oligomeric rearrangements in vitro, such that at physiological pH values above 6.7 it predominantly adopts an inactive tetrameric/oligomeric state composed of two or more dimers linked via the N-terminal inhibitory region^52,55^ (Fig. 5D). Consistent with this in vitro model, intralinks detected within ATIF1 in wild-type mitochondria spanned the entire protein sequence (Fig. 5E), indicating that both the N-terminal inhibitory and C-terminal dimerization regions participate in intermolecular interactions within ATIF1 oligomers. In contrast, in *Lrpprc* knockout mitochondria, the intralink distribution shifted substantially toward the C-terminal dimerization region, leaving the N-terminal inhibitory region available for binding to the F_1_ domain of ATP synthase to suppress its catalytic activity (Fig. 5E). This interpretation is further supported by the distribution of the interlinks across the ATIF1 protein sequence. In the absence of LRPPRC, ATIF1 no longer forms higher-order oligomers but instead uses its N-terminal inhibitor region to bind directly to the ATP synthase subunits ATPA, ATPB, and ATPG (Fig. 5B and Supplementary Fig. 9). The interaction between the N-terminal inhibitory region of ATIF1 and ATP synthase was not detected in wild-type mitochondria, suggesting that this binding occurs uniquely under conditions of ATP synthase dysfunction or instability to regulate its reverse, ATP-hydrolytic mode.

Additionally, interlinks between the C-terminal dimerization region of ATIF1 and metabolic enzymes, such as MDHM, SDHA and CSIY, were also detected, providing additional evidence for their increased association with ATP synthase in *Lrpprc* knockout mitochondria (Fig. 3A–C). Together, these findings demonstrate that in *Lrpprc* knockout hearts, both ATIF1-mediated inhibition and the broader interactome remodeling occur within the F_1_ catalytic domain of ATP synthase.

### Structural reorganization of ATP synthase in the *Lrpprc* knockout hearts

**Fig. 6:**
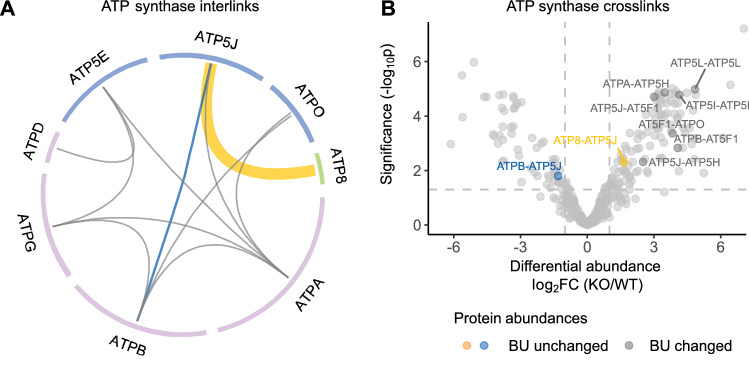
Changes in ATP synthase inter- and intralinks upon loss of *Lrpprc* in the heart. **A** Chord diagram representing the observed interlinks within the ATP synthase. The interactions significantly upregulated in the *Lrpprc* knockout are colored in yellow, and those downregulated are colored in blue. The thickness of the line is proportional to the observed fold change. Proteins are color-coded based on their location in the ATP synthase structure: F_1_ domain (pink), F_O_ domain (blue) and peripheral stalk (green). **B** Differential abundance analysis of the ATP synthase crosslinks detected by XL-MS in mitochondria of *Lrpprc* knockout hearts (KO) in comparison with wild-type (WT) hearts. The y-axis represents the -log10 *p*-value and the x-axis the log_2_ fold change (FC). The fold change was calculated as the difference in crosslink intensity between the *Lrpprc* knockout and wild-type heart mitochondria. Crosslinks involving proteins without significantly changed protein abundances according to the bottom-up (BU) proteomics analysis are highlighted in yellow and blue. Crosslinks colored in grey are between subunits that also have increased protein abundances according to the BU proteomics analysis. The annotated crosslinks have an FDR < 5% and |log_2_FC| > 1. FDR was calculated by a “moderated t-statistic” two-sided test followed by Benjamini–Hochberg correction. Number of biological replicates, *n* = 3. Source data are provided as a Source data file.

Beyond mapping interaction changes, the in situ XL-MS dataset also allowed us to examine detailed structural rearrangements within ATP synthase in the absence of LRPPRC. Analyses of intralinks within the ATP synthase revealed no significant changes among the F_1_ subunits. In contrast, loss of LRPPRC led to an increase in intralinks of the ATP5I and ATP5L subunits (Fig. 6), which are known to play a key role in ATP synthase dimerization^52^. Importantly, the ATP5I and ATP5L were also increased in abundance in our bottom-up proteomics dataset (Fig. 2A), indicating that the elevated ATP5I/ATP5L intralinks could partly reflect higher protein levels rather than structural remodeling per se. With this caveat, the elevated intralinks of the F_O_ membrane domain may reflect strengthened interactions between the ATP5I and ATP5L subunits of adjacent ATP synthase monomers, possibly acting as a compensatory mechanism to counteract dimer instability (Figs. 2C and 3D). Alternatively, the increased intralinks within these subunits could result from structural rearrangements within the F_O_ region caused by ATP6 downregulation (Fig. 2A). Furthermore, analyses of the interlinks involving ATP synthase subunits whose abundance was unchanged in bottom-up proteomics revealed increased crosslinks between F_O_ membrane subunit ATP8 and the peripheral stalk subunit ATP5J, accompanied by decreased crosslinks between F_1_ head subunit ATPB and the same peripheral stalk subunit in the absence of LRPPRC (Fig. 6). These changes indicate strengthened interactions between the F_O_ and peripheral stalk regions and weakened contacts between the F_1_ head and the peripheral stalk, in line with partial detachment of the F₁ domain from the remaining ATP synthase structure in the *Lrpprc* knockout hearts (Figs. 3D and 4)^24^. Collectively, our data show that loss of LRPPRC leads to reduced ATP6 levels, which in turn perturbs the F_O_ region of ATP synthase. This perturbation appears to trigger a compensatory increase in other F_O_ subunits, strengthening their interactions with each other and the peripheral stalk, but weakening the coupling between the F_1_ head and the rest of the complex. Together, these changes destabilize the ATP synthase and lead to the emergence of subassembled F_1_ species.

## Discussion

Mitochondrial ATP synthase is well recognized for its dual role in driving ATP production and maintaining cristae architecture. Here, we show that it is not merely a structural and catalytic component of the OXPHOS system but may also act as a dynamic hub that links mitochondrial bioenergetics with central carbon metabolism. Using in situ XL-MS on intact mitochondria combined with quantitative proteomics, BN-PAGE and complexome profiling, we demonstrate that ATP synthase directly associates with enzymes of the TCA cycle in the mouse heart and undergoes extensive structural, regulatory and interactome remodeling when its function and stability are compromised by impaired mitochondrial gene expression.

Under normal physiological conditions, the F_1_ catalytic head of ATP synthase forms extensive contacts with multiple TCA enzymes, including citrate synthase, isocitrate dehydrogenases, oxoglutarate dehydrogenase, succinyl-CoA ligase, fumarate hydratase and malate dehydrogenase within intact heart mitochondria, (Fig. 1A and Supplementary Fig. 1). Cross-species reanalysis of XL-MS data from bovine heart and human HEK293T mitochondria further validated that the observed ATP synthase-TCA cycle association patterns are conserved, at least across all these three mammalian systems^56–59^.

Together, these findings establish a previously unappreciated structural link between the OXPHOS and the TCA cycle that goes beyond complex II, which has been considered to be the sole bridge between the two pathways.

In vitro studies have shown that TCA cycle enzymes can organize into dynamic multi-enzyme assemblies or metabolon-like structures that mediate substrate channeling and reorganize in response to substrate availability and the metabolic state^54^. The physiological significance of such assemblies remains debated, with a recent review proposing that they may form transiently to optimize metabolic efficiency under conditions of high energy demand^55^. Consistent with a potential crosstalk between central carbon metabolism and respiratory chain activity, MDHM-generated oxaloacetate was shown to inhibit complex II of the respiratory chain to regulate^60^. Our findings raise the possibility that ATP synthase itself could participate in such larger metabolon-like networks in cardiac mitochondria, possibly linking local metabolic flux directly to ATP synthesis.

**Fig. 7:**
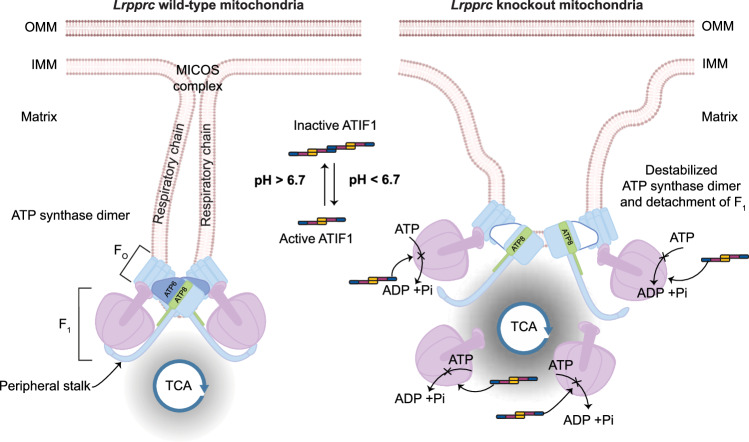
Graphical model of ATP synthase organization, its interactome and its regulatory remodeling in wild-type and *Lrpprc* knockout heart mitochondria. In wild-type heart mitochondria, ATP synthase dimers are positioned at the tips of the inner mitochondrial membrane (IMM) cristae. The complex consists of the membrane-embedded F_0_ domain, which contains the only mtDNA-encoded subunits (ATP6 and ATP8) and the matrix-exposed F_1_ catalytic head, connected via the peripheral stalk. In LRPPRC knockout heart mitochondria, reduced ATP6 levels trigger compensatory rearrangements within the F_0_ domain that ultimately destabilize the entire complex and promote partial detachment of the F_1_ head. This structural destabilization, in turn, perturbs cristae membrane organization^24^. Under physiological conditions, the endogenous ATP synthase inhibitor (ATIF1) exists predominantly as tetramers or higher-order oligomers formed by N-terminally mediated dimer-dimer interactions. In LRPPRC-deficient heart mitochondria, matrix acidification caused by respiratory chain dysfunction promotes the dissociation of ATIF1 oligomers into active dimers, exposing the N-terminal-inhibitory domain, which then binds to the F_1_ catalytic domain and inhibits ATP hydrolysis. The TCA cycle is depicted in proximity to ATP synthase, with light gray shading indicating the prominent cross-links identified between the F_1_ head and TCA cycle enzymes in wild-type mitochondria. Darker gray shading represents the further increase in crosslink abundance among the F_1_ head, ATIF1, and TCA cycle enzymes in LRPPRC knockout heart mitochondria as compared to the wild type. For completeness, the location of the MICOS complex is shown in wild-type mitochondria, as well as the location of respiratory chain complexes along the planar regions of cristae membranes. OMM-outer mitochondrial membrane. Created in BioRender. Misic, J. (2026) https://BioRender.com/zmw1fa0.

When mitochondrial gene expression is disrupted, as in the heart- and skeletal-muscle-specific *Lrpprc* knockout model used here, the ATP synthase undergoes a profound secondary structural remodeling, as dissected by complexome profiling (Fig. 2C), which ultimately perturbs the cristae morphology^24^ (Fig. 7). The selective downregulation of mtDNA-encoded subunits, notably ATP6, destabilizes the F_O_ domain of ATP synthase, triggering compensatory upregulation of other F_O_ subunits (ATP5I and ATP5L) and suggesting strengthened interactions between them (Figs. 2A and 5). In contrast, F_1_ and peripheral stalk subunits remain unchanged or are moderately upregulated (Fig. 2A). We also detected stronger interactions between F_O_ and the peripheral stalk, accompanied by weakened association between the F_1_ head and the rest of the complex. The observed compositional imbalance, together with altered intra- and inter-subunit contacts, promotes partial disassembly of the complex and accumulation of soluble F_1_ subcomplexes in the *Lrpprc* knockouts (Figs. 2C–E and 7). These findings are consistent with a defect in the late stages of ATP synthase assembly, during which the incorporation of mtDNA-encoded ATP6 and ATP8 into the F_O_ membrane domain is required to anchor the F_1_ head and stabilize the dimeric form of the complex^14^. Similarly, patients carrying mutations in ATP6 or ATP8 often display loss of dimeric ATP synthase forms and accumulation of F_1_ subspecies^61–63^. Previous studies have shown that the incorporation of peripheral stalk subunit ATP5J precedes the integration of ATP6 and ATP8^64^. In the *Lrpprc* knockout hearts, ATP5J upregulation and its strengthened interaction with ATP8 (Fig. 6) likely represent an adaptive adjustment of the assembly pathway to partially stabilize the F_O_ domain in response to ATP6 deficiency. Together, the reinforced interactions observed among some F_O_ subunits and their increased contacts with the peripheral stalk may preserve partial structural integrity and prevent complete dimer dissociation in the absence of LRPPRC. Meanwhile, the released F_1_ assemblies engage in enhanced interactions with the enzymes of the TCA cycle, fatty-acid β-oxidation and ketone-body metabolism, as revealed by XL-MS (Fig. 3C and Supplementary Data 2).

Our BN-PAGE analyses further corroborate the in situ XL-MS findings, confirming the reorganization of ATP synthase and its altered interactions in the *Lrpprc* knockout hearts (Figs. 3D and 4). The F_1_ subunit ATPA co-migrates with MDHM in *Lrpprc* knockouts as well as in other models of impaired mtDNA maintenance and expression in the heart, such as *Tfam* and *Rnaseh1* knockouts. These findings suggest that the enhanced association between the F_1_ head and metabolic enzymes, exemplified here by MDHM, likely represents a general feature that emerges under conditions of defective mtDNA expression and subsequent ATP synthase dysfunction and instability. Further supporting our data, a complexome profiling study of wild-type heart mitochondria also detected co-migration of ATPA, ATPB, MDHM, and CISY at comparable molecular weights (reanalyzed data in Supplementary Fig. 10)^65^, indicating that these associations are present at a basal level under normal physiological conditions and are therefore captured in our XL-MS dataset of wild-type hearts (Fig. 1 and Supplementary Fig. 1). In our BN-PAGE analyses, co-migration became detectable only under conditions of ATP synthase dysfunction, likely because the interactions become more abundant upon structural destabilization of the enzyme, as evidenced by the increased crosslink intensities observed in the *Lrpprc* knockout hearts (Fig. 3A–C).

We also identify a regulatory remodeling of ATP synthase mediated by ATIF1. The *Lrpprc* knockout mitochondria yielded increased crosslinks of ATIF1 to the F₁ head, as well as a redistribution of ATIF1 toward detached F_1_ (Figs. 2C and 5C). ATIF1 has been reported to undergo pH-dependent changes affecting the oligomerization state^16,55^ (Fig. 7). Our XL-MS data provide in vivo evidence that ATIF1 transitions from inactive oligomers to inhibitory dimers upon activation, which leads to the N-terminal inhibitory region engaging with the catalytic subunits of the F_1_ domain in *Lrpprc* knockouts. Consistently, our complexome profiling data support the association of ATIF1 to the F_1_ head. This conformational switch prevents wasteful ATP hydrolysis by effectively shifting the enzyme from an energy-producing to an energy-preserving state. Our findings are consistent with previous reports showing that ATIF1 activity is also modulated by PKA-dependent phosphorylation at Ser39 within the N-inhibitory domain. Phosphorylation at this site prevents ATIF1-binding to F_1_ under normal conditions^60^. Notably, we also detected crosslinks between ATIF1 and TCA cycle enzymes in the *Lrpprc* knockouts (Supplementary Fig. 9), suggesting that ATP synthase inhibition may be integrated with a broader metabolic remodeling program in mitochondria. In summary, our findings reveal a coordinated structural and interactome remodeling of ATP synthase that integrates metabolic, architectural and inhibitory mechanisms. Under basal conditions, ATP synthase shows substantial in situ contacts with multiple TCA cycle enzymes, suggesting that it may reside at a spatial interface between OXPHOS and central carbon metabolism. Under mitochondrial dysfunction caused by impaired mtDNA gene expression, ATP synthase becomes structurally destabilized, detached F_1_ accumulates and shows increased association with metabolic enzymes, accompanied by redistribution of its intrinsic inhibitor ATIF1 toward F_1_-containing species. We hypothesize that this remodeling reflects an adaptive reorganization of matrix metabolism under respiratory chain limitation and redox stress. Respiratory chain limitation increases the NADH/NAD^+^ ratio^66^, which can constrain oxidative TCA cycle reactions and may promote redox-adaptive shifts in enzyme directionality. For example, in cells that lack mitochondrial respiration (ρ0 cells), MDHM was shown to switch direction and convert oxaloacetate to malate to help rebalance the NADH/NAD^+^ ratio^67^. Similarly, in vivo isotope-tracing studies in clear cell renal cell carcinoma showed that reduced respiratory capacity is associated with slower oxidative TCA-cycle turnover^68^.

Taken together, the results we present here may hold broad relevance for mitochondrial pathophysiology. Defects in ATP synthase assembly or mtDNA expression underlie a spectrum of human disorders, including mitochondrial encephalomyopathies, cardiomyopathies and other systemic manifestations^29^. Future work will be required to define the functional consequences of this metabolic remodeling and to test whether it can be therapeutically targeted in conditions involving impaired ATP synthase activity or stability.

The data presented in this study reveal extensive and intricate interactions between ATP synthase and multiple TCA cycle enzymes in situ and how they are remodeled when ATP synthase function and integrity are compromised. Future functional studies with biochemical and genetic manipulations will be required to define the metabolic consequences of these interactions.

In addition, the quantitative XL-MS workflow introduced in this study provides opportunities for the crosslinking community. Further refinement of the data filtering procedures and implementation of more advanced normalization strategies and isotopically labeled crosslinkers may enable the recovery of and more precise quantification of additional protein interactions, addressing persistent challenges in XL-MS.

## Methods

### Animals and housing

Heart- and skeletal muscle-specific *Lrpprc*, *Tfam,* and *Rnaseh1* knockout mice were generated by crossing mice carrying floxed alleles of the respective genes with transgenic mice expressing Cre recombinase under the control of the muscle creatine kinase promoter (Ckmm*-Cre*). All lines were maintained on a C57BL/6N background and have been reported previously^43,47,48^. For all experiments, mice were euthanized by cervical dislocation, and hearts were collected at the latest viable humane endpoint ages for each model (10–12 weeks of age for *Lrpprc* knockouts, 7–9 weeks of age for *Tfam* knockouts and 24 weeks for *Rnaseh1* knockouts). Both male and female mice were included where available, and age-matched data were pooled across sexes. The study was designed to assess genotype-dependent molecular phenotypes and was not powered for sex-specific analyses. Animals were maintained in individually ventilated cages under a 12-h light/dark cycle at an ambient temperature of 22 ± 2 °C and relative humidity of 50 ± 10%, with ad libitum access to chow and water. All procedures were approved by the Stockholm research animal ethics committee under ethical permit number 18936-2022 and carried out in accordance with national and European legislation.

### Mitochondria isolation

For in situ crosslinking, intact mitochondria were prepared in isolation buffer (MIB) containing 200 mM sucrose, 20 mM HEPES, and 1 mM EDTA. For all other purposes, mitochondria were prepared in MIB consisting of 235 mM sucrose, 20 mM Tris-HCl (pH 7.4), and 1 mM EDTA. Hearts were excised after cervical dislocation and transferred into ice-cold PBS. After rinsing to remove blood, hearts were minced on ice and transferred into 1.8 mL of MIB supplemented with 200 µL of 2.5% trypsin. Samples were rotated at 4 °C for 10 min, diluted with MIB containing 0.04% trypsin inhibitor, and homogenized. The homogenate was centrifuged at 1000 × *g* for 10 min at 4 °C, and the supernatant was collected and centrifuged at 10,000 × *g* for 10 min. The resulting pellet was resuspended in MIB containing 0.2% BSA, adjusted to 12 mL, gently mixed, and centrifuged again at 10,000 × *g*. The final pellet was washed twice with MIB lacking BSA and trypsin inhibitor, then resuspended in 150 µL of MIB. All steps were carried out on ice or at 4 °C. Mitochondrial protein concentration was determined using a Qubit fluorometer (Invitrogen).

### BN-PAGE

BN-PAGE was performed following the general principles of established BN-PAGE methodology^4^ using modifications described previously^5^. Mitochondrial proteins (75 µg) were incubated on ice in 50 µL of buffer containing 20 mM Tris-HCl (pH 7.4), 50 mM NaCl, 0.1 mM EDTA, and 10% (v/v) glycerol. Proteins were solubilized with 1–3% (w/v) digitonin (Calbiochem), after which native loading dye (5% Coomassie Brilliant Blue G-250, 150 mM Bis-Tris, 500 mM ε-aminocaproic acid, pH 7.0) was added. Samples were subsequently loaded onto in-house prepared 3–13% gradient BN-PAGE gels for separation of protein complexes.

### ATPase in gel activity assay

To visualize ATPase activity, BN-PAGE gels were incubated in a solution containing 50 mM glycine, 5 mM MgCl_2_, 0.1% Triton X-100, and 0.5 mg/mL lead nitrate, adjusted to pH 8.4. After an initial 30 min incubation in 50 mL of this buffer, an additional 50 mL of the same buffer supplemented with 4 mM ATP was added, followed by a 1 h incubation. The gels were then rinsed with water and scanned against a dark background.

### Western blotting

Proteins separated by SDS-PAGE or BN-PAGE were transferred to Hybond polyvinylidene fluoride (PVDF; GE Healthcare) membranes. For fluorescence-based detection, membranes were blocked in Intercept Blocking Buffer (LI-COR), incubated with a mixture of primary antibodies, and probed with a mixture of donkey anti-mouse IgG 800CW and goat anti-rabbit IgG 680RD secondary antibodies (LI-COR). Fluorescent signals were visualized using the LI-COR Odyssey imaging system. Chemiluminescent detection was performed using Clarity Western ECL Substrate (Bio-Rad). Primary antibodies included anti-ATP5A (Abcam, ab14748), anti-MDH2 (Abcam, ab96193), anti-SDHA (Abcam, ab14715), and anti-CS (Santa Cruz, sc-390693), each used at a dilution factor of 1:1000.

### Optimization of crosslinker concentration

The concentration of DSSO was optimized as previously described^69^. Briefly, intact mouse heart mitochondria purified from three biological replicates of wild-type and *Lrpprc* knockout mice were treated with 0.25, 0.5, or 1 mM DSSO for 45 min at 15 °C and compared to control samples incubated at either 4 or 15 °C. Subsequently, the reaction was quenched for 30 min at 15 °C, and the crosslinked samples were visualized by SDS-PAGE.

### Crosslinking of mouse heart mitochondria

Intact mouse heart mitochondria (500 µg) purified from three biological replicates of wild-type and *Lrpprc* knockout mice were treated with 0.5 mM DSSO (CF Plus Chemicals) for 45 min at 15 °C, followed by quenching for 30 min at 15 °C. Samples were processed as previously described^5^. Briefly, crosslinked mitochondria were pelleted at 11,000 × *g* at 4 °C for 10 min and resuspended in 5 times the volume of lysis buffer (7 M Urea, 1% [v/v] Triton-X-100, 100 mM Tris pH 8.5, 5 mM tris(2-carboxyethyl)phosphine (TCEP), 30 mM chloroacetamide (CAA), proteinase inhibitor cocktail, 1% [vol/vol] benzonase and 2 mM Mg^2+^). Mitochondria were solubilized for 30 min on ice and centrifuged at 18,000 × *g* for 30 min. The supernatant was collected, 1% benzonase (v/v) was added, and the sample was incubated for 2 h at room temperature while shaking. The mitochondrial proteins were precipitated following the Methanol/Chloroform precipitation protocol as previously described^70^. The dry protein pellet was resuspended in digestion buffer (1% sodium deoxycholate [w/v], 100 mM Tris pH 8.5, 5 mM TCEP and 30 mM CAA) to a final protein concentration of 1 µg/µL. Protein digestion was performed first with LysC (1:100 enzyme to protein ratio) for 45 min at 37 °C, followed by overnight digestion with trypsin (1:25 enzyme to protein ratio) at 37 °C. Digestion was stopped by the addition of trifluoroacetic acid (TFA) to a final concentration of 0.5% (v/v) TFA. Samples were centrifuged at 18,000 × *g* at 4 °C for 10 min and the supernatant was collected.

Finally, peptides were desalted using solid-phase extraction C18 columns (Sep-Pak, Waters) and fractionated into 27 fractions for DSSO using an Agilent 1200 HPLC pump system (Agilent) coupled to a strong cation exchange (SCX) separation column (Luna SCX 5 mm to 100 Å particles, 50 × 2 mm, Phenomenex). The fractions were desalted using Oasis.

### Complexome profiling

Samples of wild-type and *Lrpprc* knockout mitochondria purified from mouse heart were run on BN-PAGE in triplicate, and each lane was cut into 24 equal-sized bands with a sharp scalpel and further chopped into small pieces. The gel pieces were subjected to in-gel digestion^71^. Briefly, the gel pieces were washed and reduced by incubation in reduction buffer (6.5 mM DTT, 50 mM AMBIC, pH 8.5) for 1 h at 60 °C. The reduction buffer was removed, the gel pieces were dehydrated with 100% ACN, and alkylated by incubation in alkylation buffer (55 mM IAA, 50 mM AMBIC, pH 8.5) for 30 min at room temperature. The alkylation buffer was removed, the gel pieces were dehydrated with 100% ACN and subsequently washed two times with 50 mM AMBIC and 100% ACN. The dehydrated gel pieces were incubated in cold trypsin solution (3 ng/μL in 50 mM AMBIC cold, pH 8.5) for 90 min on ice, the excess of trypsin was removed, and the gel pieces were incubated overnight at 37 °C in 50 mM AMBIC. The supernatant was collected, the gel pieces were dehydrated with 100% ACN, and both supernatants were combined.

### LC-MS/MS analysis

The fractionated samples were analyzed using a Exploris 480 (ThermoFisher) mass spectrometer coupled to an Ultimate3000 UHPLC system (ThermoFisher). The fractions were injected at 3 µL/min for 1 min into a trap column (Acclaim Pepmap 100 C18, 5 mm × 0.3 mm, 5 µm, Thermo Fisher Scientific). The peptide separation was performed in a 50 cm long analytical column with a 75 µm inner diameter packed in-house with C18 beads (Reprosil C18, 1.9 µm) at a column temperature of 32 °C. Peptides were eluted at 300 nL/min with a total run time of 70 min, using a gradient with solvent A (0.1% (v/v) formic acid) and solvent B (80% (v/v) acetonitrile and 0.1% (v/v) formic acid). Gradient separation on the analytical column: 9% B for 1 min, from 9 to 13% B in 1 min, from 13 to 41% in 55 min, from 41 to 99% in 1 min, 99% B for 4 min, 99 to 9% in 1 min and 9% B for 7 min. The MS acquisition method was a data-dependent acquisition mode using the Orbitrap analyzer at 60 K mass resolution in the scan range 375–2200 m/z, with standard AGC target and automatic maximum injection time. Ions with charges between +3 and +8 were selected for MS2 and filtered based on dynamic exclusion with a mass tolerance of 10 ppm and mass resolution of 30 K. Peptides were fragmented with stepped HCD of 19, 25, and 28%.

For the complexome profiling samples, the same LC-MS/MS setup and parameters were used as described above, unless otherwise indicated. The total run time was 60 min with gradient separation on the analytical column: 4% B for 1 min, from 4 to 11% B in 2 min, from 11 to 30% in 32 min, from 30 to 44% in 5 min, from 44 to 55% in 4 min, from 55 to 99% in 1 min and 99% B for 4 min, and 4% B for 10 min. The MS acquisition method was a data-independent acquisition (DIA) mode using the Orbitrap analyzer at 60 K mass resolution in the scan range 375–1600 m/z. The DIA experiment was acquired with a precursor mass range within 400–1000 m/z with a 20 m/z isolation window. Peptides were fragmented with HCD 28%.

### MS data analysis

For proteomics analysis, the raw files corresponding to all fractions were analyzed with MaxQuant (version 2.4.14.0) using the full *Mus musculus* reference proteome (downloaded from UniProt 15/02/2024 with UniProtID: UP000000589). Tryptic digestion with a maximum of three misscleavages was selected. Carbamidomethyl (C) was set as a static modification, and oxidation (M) and protein N-terminal acetylation as variable modifications. Non-crosslinked peptides were quantified based on the sum of protein intensity across the different fractions.

For the DSSO XL-MS experiments, a FASTA file containing all identified proteins across both XL-MS experiments was generated from the proteomics search; the mitochondrial transit peptides were annotated with TargetP 2.0^72^ and subsequently removed. The XL-MS data were analyzed in Proteome Discoverer with the XlinkX node for analysis of crosslinked peptides, as previously reported^69^. For the XlinkX search, tryptic digestion with a maximum of three misscleavages, 10 ppm error for MS1 and 20 ppm error for MS2. Carbamidomethyl (C) was set as a static modification, and oxidation (M) and protein N-terminal acetylation as variable modifications. The MS1 intensity information was retrieved from the MaxQuant table “allPeptides.txt,” previously generated in the proteomics search. The crosslink identifications extracted from the XlinkX “PSMs” table were combined with the feature MS1 intensities from the MaxQuant “allPeptides.txt” table, based on the raw file name and the MSMS scan number. Therefore, generating a table containing all the XL-MS information from XlinkX and the MS1 intensities from MaxQuant. Crosslinked peptides were accepted with XlinkX score higher or equal to 40 and a maximum FDR of 5%. Crosslinks present in less than 2 replicates per sample were excluded. Crosslinked peptides were quantified by the addition of the MS1 precursor intensities across all spectral matches. Crosslinks without precursor intensities were excluded for analysis.

For complexome profiling, the raw files were analyzed with DIA-NN (version 1.8.1) using the FASTA file generated for the crosslinking experiments and known contaminants. Library-free search was performed with tryptic digestion with a maximum of two missed cleavages and mass accuracy of 20 ppm. Carbamidomethyl (C) was set as a static modification, and oxidation (M) as a variable modification. The maximum FDR was set to 1%.

### Quantification of changes in proteins and protein–protein interactions

Data analysis and visualization were performed with custom R scripts. Data were analyzed in R version 4.4.0 running in RStudio 2024.12.1 (Build 563). Data handling and visualization were mostly performed with *tidyverse* (v2.0.0), *protti* (v0.8.0), and *cowplot* (v1.1.3).

For bottom-up proteomics, protein precursor intensities were median-normalized and log_2_-transformed. Imputation was performed based on the “Ludovic” method, followed by differential abundance analysis with a “moderated t-test” using the *protti* package in R. Differential abundance of the proteins was defined as significant by adjusted *p*-value < 0.05 and |log_2_ fold change| > 1.

For XL-MS, crosslink precursor intensities were median-normalized and log_2_-transformed. Imputation was performed as described for the bottom-up proteomics analysis. Differential abundance of the crosslinks was defined as significant by adjusted *p*-value < 0.05 and |log_2_ fold change| > 1.

For complexome profiling, median protein intensities across replicates were calculated and then summed for each slice based on the ATP synthase substructures (F_1_, Fo, and peripheral stalk) and ATIF1. The summed intensities were normalized to the maximum intensity per lane separately for each group.

### Reporting summary

Further information on research design is available in the Nature Portfolio Reporting Summary linked to this article.

## Supplementary information

Supplementary Information

Description of Additional Supplementary Files

Supplementary Data 1

Supplementary Data 2

Reporting Summary

Transparent Peer Review File

## Source data

Source Data

## Data Availability

The proteomics and crosslinking mass spectrometry data have been deposited in the ProteomeXchange Consortium via PRIDE^73^ partner repository with the dataset identifier PXD071441 http://proteomecentral.proteomexchange.org/cgi/GetDataset?ID=PXD071441. The complexome profiling data were deposited under the dataset identifier PXD075583 http://proteomecentral.proteomexchange.org/cgi/GetDataset?ID=PXD075583. Source data are provided with this manuscript. Source data are provided with this paper.
