# Supplementary material for: Investigating the relationship between ATP synthase and the TCA cycle by crosslinking mass spectrometry
Source: Nat Commun. 2026 Jun 23;17:5563. doi: 10.1038/s41467-026-74730-5 (PMC13291236; doi:10.1038/s41467-026-74730-5)
Supplement: Supplementary file 1 — Supplementary Information [file 41467_2026_74730_MOESM1_ESM.pdf]

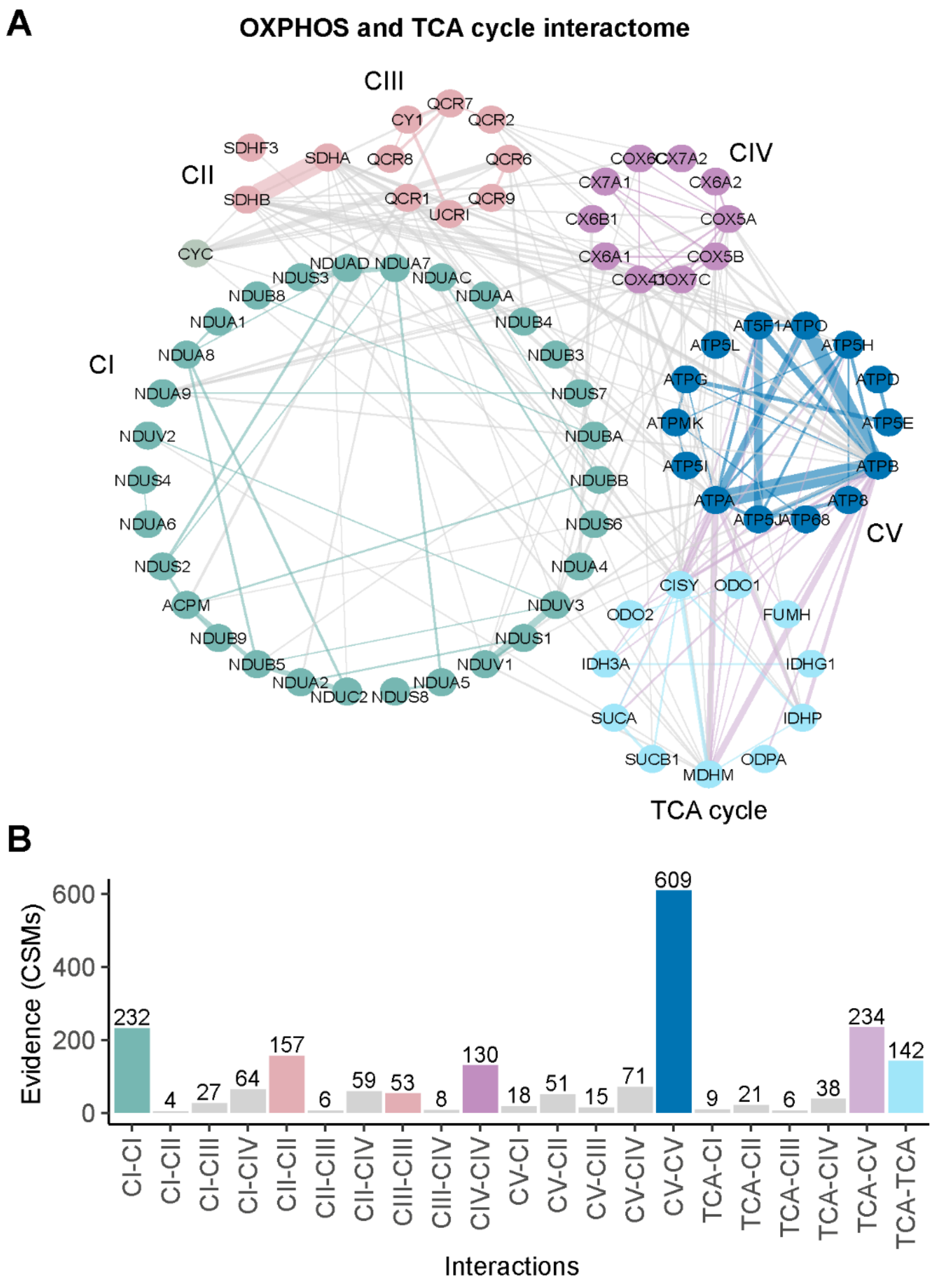

Supplementary Fig. 1. XL-MS provides evidence for extensive interactions within and between the OXPHOS and TCA cycle proteins in mouse heart mitochondria. (Based on data from Milenkovic *et al* [5]).

(A) Overview of protein interactions within the OXPHOS complexes and the TCA cycle, the names are abbreviated as follows: complex I (CI), complex II (CII), complex III (CIII), complex IV (CIV), ATP synthase (CV) and TCA cycle (TCA). (B) Bar plot of crosslink spectral matches (CSMs) detected between and within complexes across n=3 biological replicates.

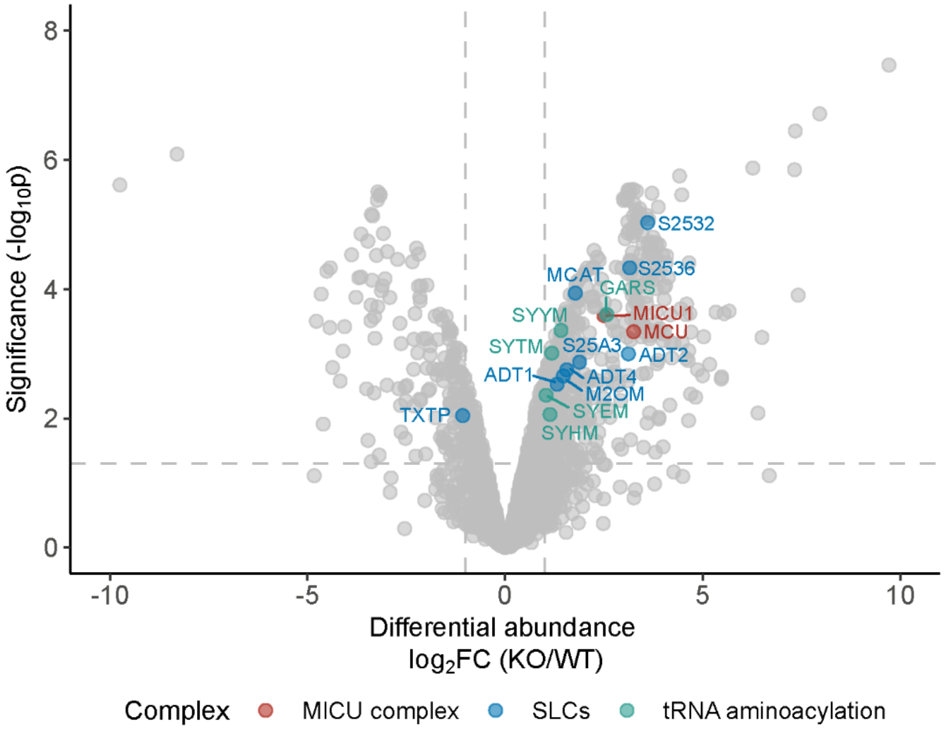

Supplementary Fig. 2. Volcano plot highlighting previously undetected dysregulated mitochondrial proteins in heart mitochondria of tissue-specific *Lrpprc* knockout mice. Differential expression analysis of protein abundance as detected in heart mitochondria of the tissue-specific *Lrpprc* knockout (KO) compared with wild-type (WT) mice. The y-axis represents the-$\log_{10}$ p-value and the x-axis the $\log_2$ fold change (FC). The proteins from the mitochondrial calcium uptake (MICU) complex, mitochondrial solute carriers (SLCs) and tRNA aminoacylation-related enzymes are highlighted. Thresholds are FDR < 5% and $|\log_2 FC| > 1$. The FDR was calculated by a "moderated t-statistic" two-sided test followed by Benjamini-Hochberg correction. Number of biological replicates, n=3. Source data are provided as a Source Data file.

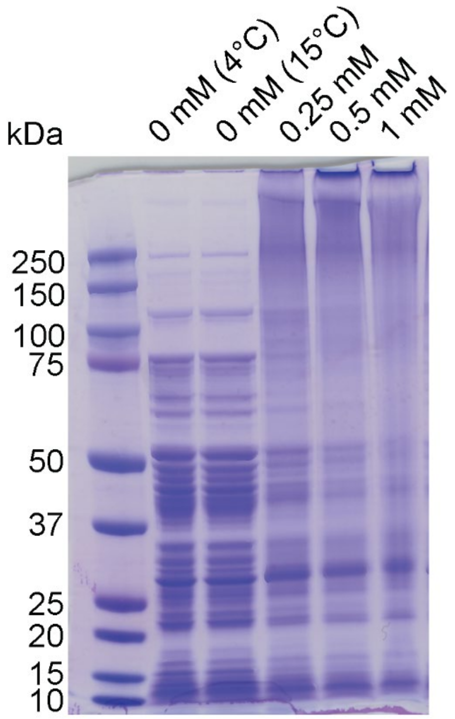

Supplementary Fig. 3. SDS-PAGE used for the optimization of crosslinker concentration for the XL-MS experiments on mouse heart mitochondria.

Different concentrations of DSSO (0.25, 0.5 and 1mM) were tested for the crosslinking reaction and 0.5 mM was selected as the optimal concentration. The control reaction was tested both at 4°C and at 15°C to account for possible changes due to incubation temperature. Each lane represents a fraction of the same sample treated as indicated. Representative gel from three independent experiments with similar results.

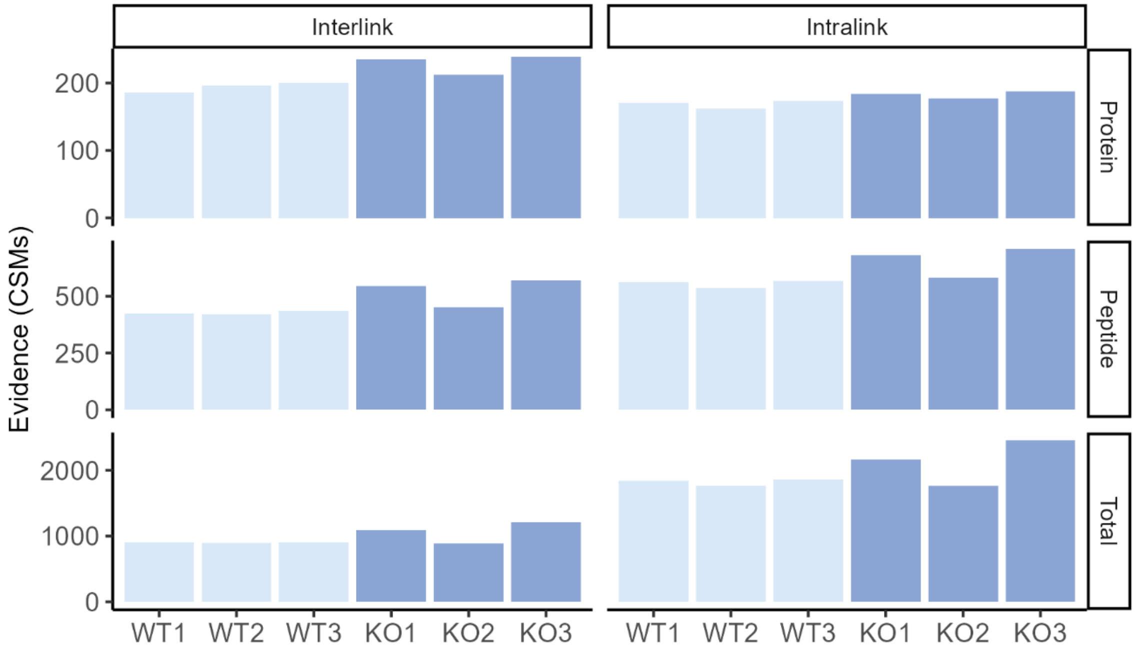

Supplementary Fig. 4. Overview of CSMs detected per replicate in the XL-MS experiments. Crosslink spectral matches (CSMs) identified per unique protein pair, per peptide pair, and in total are shown. The values show good reproducibility across n=3 biological replicates. Source data are provided as a Source Data file.

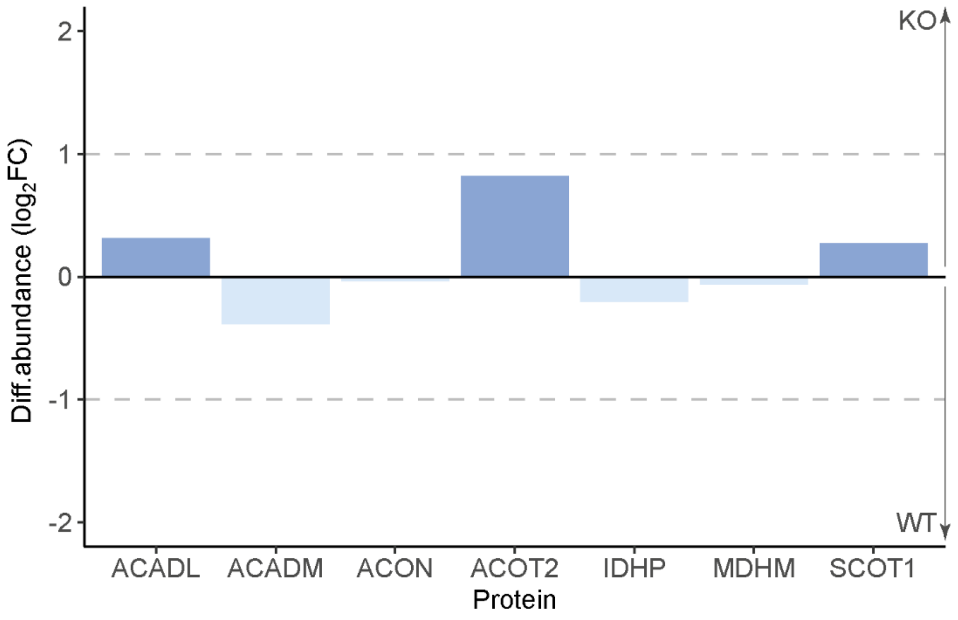

Supplementary Fig. 5. Log_2 fold change (FC) in abundance of mitochondrial metabolism proteins interacting with ATP synthase in the tissue-specific *Lrpprc* knockout hearts.

The fold change represents the difference in protein abundance between the *Lrpprc* knockout (KO) and the wild-type (WT) heart mitochondria. All proteins had FDR > 5% and $|Log_2FC| > 1$ and were therefore considered not to be significantly changed. Number of biological replicates, n=3. Source data are provided as a Source Data file.

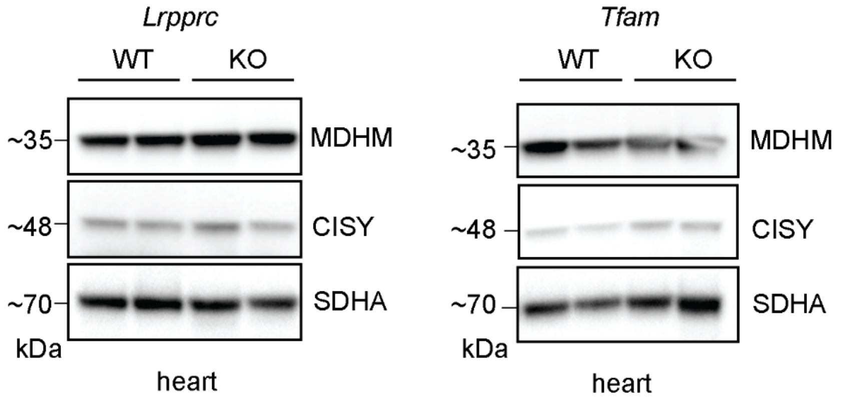

Supplementary Fig. 6. Western blot analyses of SDS-PAGE-separated heart mitochondria from tissue-specific Lrpprc and Tfam knockout (KO) and control (WT) animals.

No difference in abundance was observed in MDHM and CISY levels between the KO and WT mice. Minor differences might be caused by variability during sample loading. Each lane represents n=1 biological replicate. Representative immunoblots from at least three independent experiments with similar results. Source data are provided as a Source Data file.

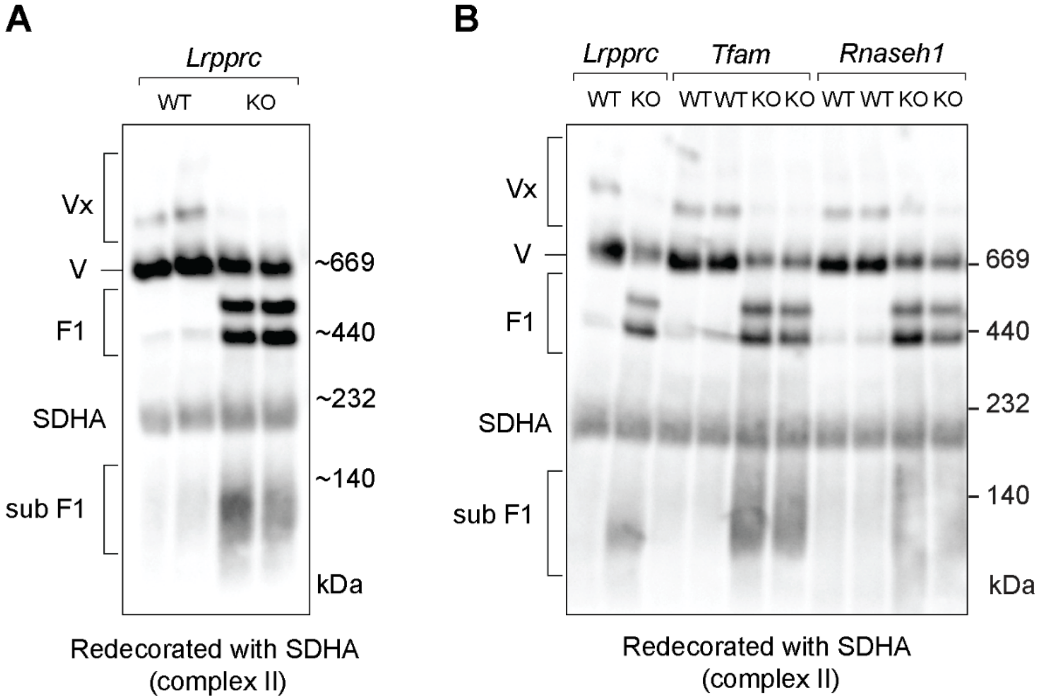

Supplementary Fig. 7. Loading control for fluorescent western blots of BN-PAGE-separated heart mitochondria from tissue-specific *Lrpprc*, *Tfam* and *Rnaseh1* knockouts (KO) and controls (WT) presented in the main figures.

Succinate dehydrogenase subunit A (SDHA, complex II) was used as the loading control for the fluorescent western blots shown in Fig. 3D (panel D) and Fig. 4B (panel B). After fluorescence imaging, the same membranes were re-incubated with anti-SDHA antibody and detected by HRP-based colorimetric scanning. Each lane represents n=1 biological replicate. Representative immunoblots from three independent experiments with similar results. Source data are provided as a Source Data file.

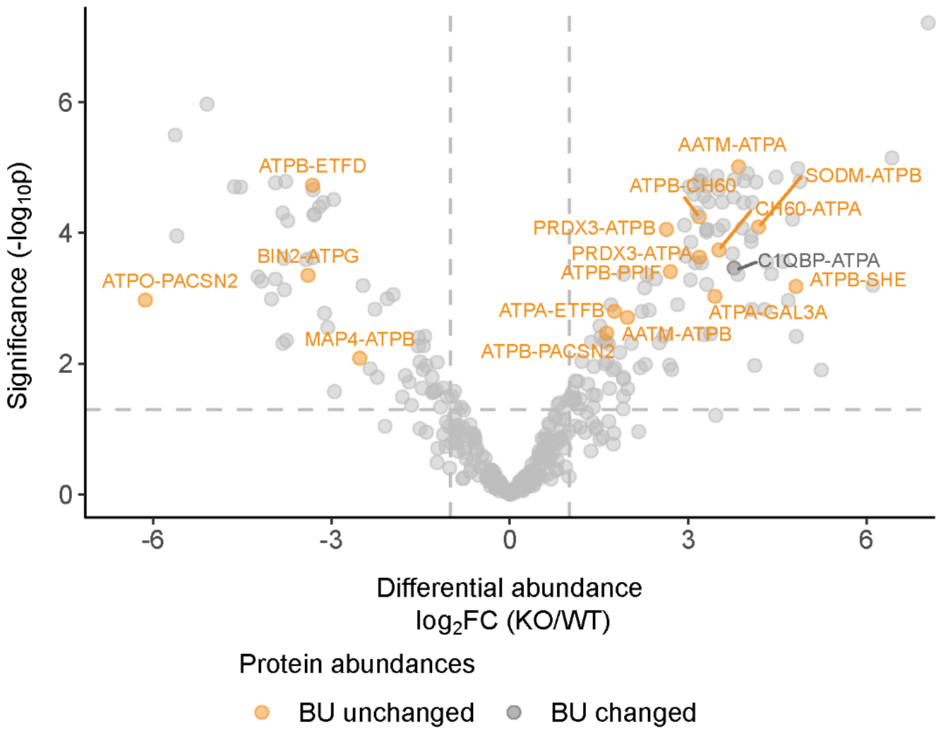

Supplementary Fig. 8. Interaction between ATP synthase and non-OXPHOS or non-TCA proteins.

Differential abundance analysis of the ATP synthase (CV) crosslinks with “other” proteins detected in the *Lrpprc* knockout (KO) compared to the wild-type (WT) heart mitochondria by XL-MS. The proteins annotated as “other” exclude all the OXPHOS structural components and TCA enzymes. The interactions with ATIF1 are also excluded as they are presented in Fig. 5A. Crosslinks involving proteins without significantly changed protein abundances according to the bottom-up (BU) proteomics analysis are highlighted in yellow; otherwise, they are colored in grey. The annotated crosslinks have an FDR < 5% and $|\log_2 FC| > 1$. The FDR was calculated by a “moderated t-statistic” two-sided test followed by Benjamini-Hochberg correction. Number of biological replicates, n=3. Source data are provided as a Source Data file.

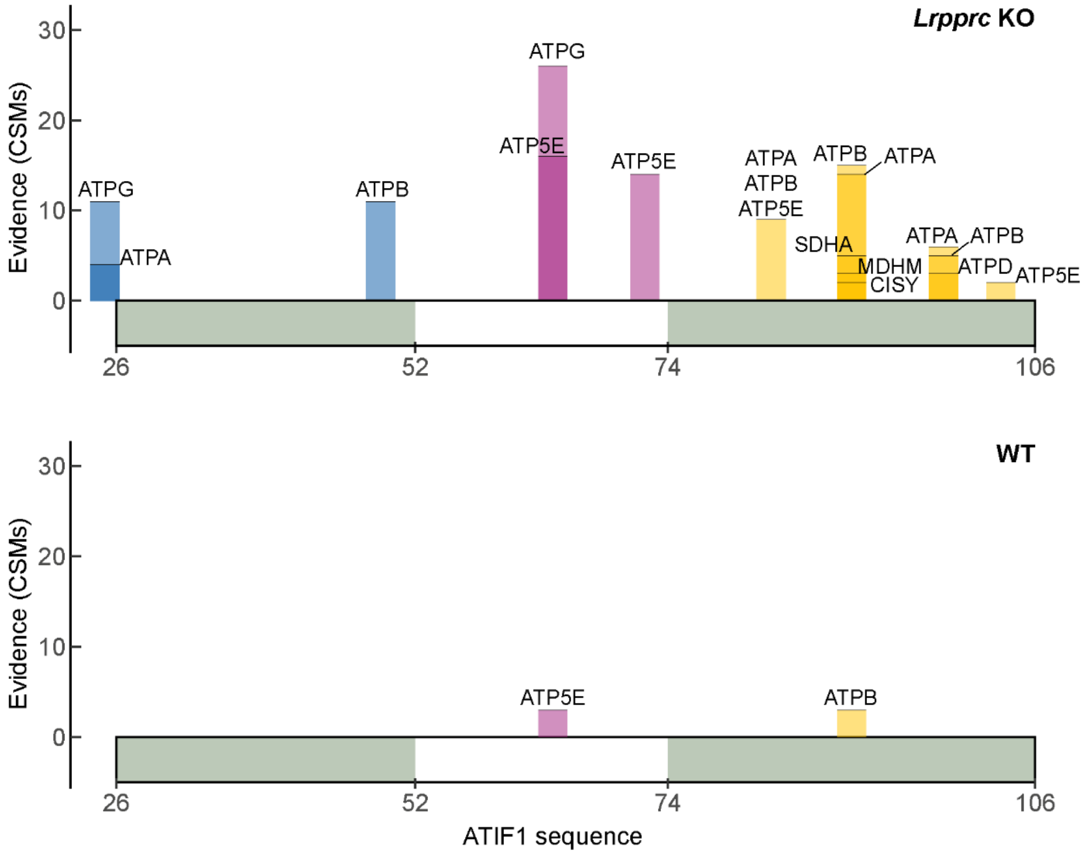

Supplementary Fig. 9. Distribution of interlinks of ATIF1 with ATP synthase proteins or metabolic enzymes.

Interlinks mapped on the amino acid sequence of ATIF1 (without the mitochondrial targeting peptide) as detected the heart mitochondria of tissue-specific *Lrpprc* knockout (KO, top) or wild-type (WT, bottom) mice. For clarification, if different proteins are located at the same position on the bar, the number of detected crosslink spectral matches (CSMs) was identical. The CSMs represent the cumulative sum across n=3 biological replicates. Source data are provided as a Source Data file.

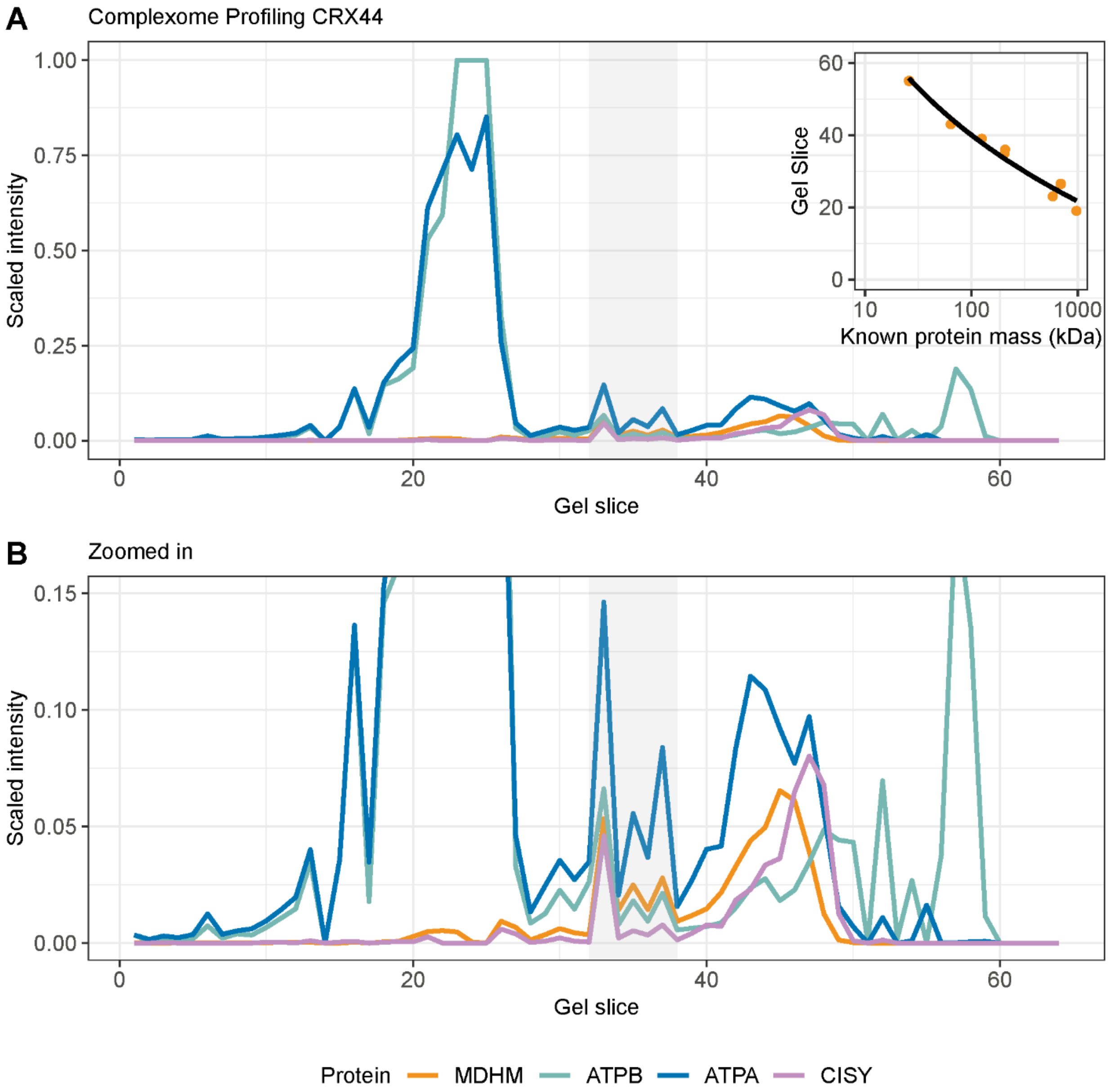

Supplementary Fig. 10. Complexome profiling of wild-type mouse heart mitochondria shows co-migration of ATPA, ATPB, MDHM and CISY.

The plot shows the protein intensities obtained by analysis of 64 gel slices by LC-MS/MS, from hig her to lower molecular weight. The low Mw form of MDHM and CISY elute at gel slices > 40. The slices wherein these proteins co-migrate with ATPB and ATPA are highlighted in grey, the estimated molecular weight is in the range of 275-140kDa. The molecular weight was estimated using the migration and masses of known complexes (see mass calibration graph). (B) shows the zoomed in view of (A). Data reanalyzed from *Yin et al* [65]; available in the CEDAR database with accession number CRX44. n=1 biological replicate.

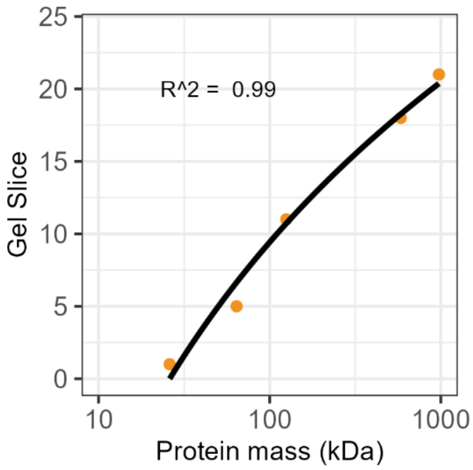

Supplementary Fig. 11. Mass calibration graph for the complexome profiling data.
The molecular weight estimations were performed using the migration and masses of known complexes. n=1.
