# Supplementary material for: Investigating the relationship between ATP synthase and the TCA cycle by crosslinking mass spectrometry
Source: Nat Commun. 2026 Jun 23;17:5563. doi: 10.1038/s41467-026-74730-5 (PMC13291236; doi:10.1038/s41467-026-74730-5)
Supplement: Supplementary file 2 — Description of Additional Supplementary Files [file 41467_2026_74730_MOESM2_ESM.pdf]

Description of Additional Supplementary Files

File Name: Supplementary Data 1

Description: Differential abundance analysis results for the bottom-up proteomics experiments

File Name: Supplementary Data 2

Description: Differential abundance analysis results for the XL-MS experiments
