# Supplementary material for: Investigating the relationship between ATP synthase and the TCA cycle by crosslinking mass spectrometry
Source: Nat Commun. 2026 Jun 23;17:5563. doi: 10.1038/s41467-026-74730-5 (PMC13291236; doi:10.1038/s41467-026-74730-5)
Supplement: Supplementary file 6 — Transparent Peer Review File [file 41467_2026_74730_MOESM6_ESM.pdf]

nature portfolio

Peer Review File

Investigating the relationship between ATP synthase and the TCA cycle by crosslinking mass spectrometry

Corresponding Author: Professor Albert Heck

This file contains all reviewer reports in order by version, followed by all author rebuttals in order by version.

Version 0:

Reviewer comments:

Reviewer #1

(Remarks to the Author)
In their study Paneda et al investigated the structural and functional integration of ATP synthase (Complex V) within the mitochondrial metabolic network. By employing in-solution crosslinking mass spectrometry (XL-MS), quantitative proteomics, and Blue Native PAGE (BN-PAGE), the authors identify a direct physical association between the F1F1 catalytic head of ATP synthase and several TCA cycle enzymes. They further demonstrate that this interaction is strengthened under mitochondrial stress (LRPPRC deficiency) and characterize the regulatory role of the ATPase inhibitory factor 1 (ATIF1).
The manuscript is well written, and conclusive. This study challenges the traditional view that Complex II is the sole structural bridge between the TCA cycle and the OXPHOS system. The discovery of a "metabolon-like" hub centered around ATP synthase is a significant conceptual advance. The observation that these interactions are dynamic and responsive to mitochondrial dysfuncton(LRPPRC, TFAM, and RNase H1 models) adds a layer of physiological relevance. That said, this study is of great interest to the mitochondrial research field and a very nice example of what can be done with XL-MS as a technique.
The following points would further strengthen the paper before publication in Nature Communications.

1. The authors compare an LRPPRC knockout to its wt analogue and investigate a change in crosslink intensity or counts. At the same time, their quantitative proteomics approach shows regulation of protein in the same direction as the detected crosslinks (i.e. in Figure 2 A vs E). The manuscript would benefit from a more explicit discussion on how they normalized crosslink intensities relative to protein abundance. While they mention using quantitative proteomics as a reference, a more formal "crosslink-to-protein ratio" analysis for key interactions would strengthen the claim that the affinity or conformation has changed, not just the concentration.

2. Functional Consequences of the ATP Synthase-TCA Link:The manuscript suggests that this link coordinates ATP synthesis with metabolic flux. While the current study is very strong on structural proteomics its lighter on metabolic flux data. Including (or citing) evidence of altered TCA cycle kinetics or substrate channeling in the LRPPRC model would bridge the gap between "physical link" and "functional coordination." For instance, does the association with MDHM specifically alter the rate of malate oxidation?

3. Figure 2D vs E: The authors describe that the ATPA-MDHM interaction has the strongest change of #CSM in KO vs wt. This is further confirmed by native PAGE western blot (Figure 2F). However, in the quantitated XL data (Figure 2E) it seems that other interactions highlighted there are regulated at much higher fold change and with a better p value. Do you have any hypothesis why the number of CSMs did not change accordingly even stronger for those candidates? Can you check the “regulation” by CSM counting at least of some of the ATP synthase-OM links as well to compare and get a better estimate of the reliability of the applied MS1 based intensity comparison for the purpose of crosslink quantification.
I think this is of special importance as crosslink quantification is done quite less frequently compared to generation of pure qualitative information in the XL-MS field. In conclusion this reflects a potential easy to apply and effective strategy for others from technical perspective. Further, other findings within this study (i.e. Figure 5) are entirely based on this quantification approach hence its reliability should be clearly shown.

4. To that end, in the methods part, the authors describe: “Crosslinked peptides were quantified by the addition of the MS1 precursor intensities across all spectral matches. Crosslinks without precursor intensities were excluded for analysis” which

makes sense, but it would be important to add more details here to allow reproducibility. For fold change calculations: in how many replicates did the crosslinks had to be quantified for being used for fold changes and volcano plots? Was any normalization or filter, next to the mentioned score threshold of 40 for XlinkX IDs, applied (i.e. a minimal signal/noise)? How was readout of MS1 quantities done, is this reported by XlinkX in PD, or was a custom script/different software used? If so, can you share the code?

5. Methods, XL of mitochondria: How was DSSO added (as stock solution at given concentration in DMSO?). Since the quality purity and hence needed optimal molarity differs a lot from vendor to vendor in my experience, for reproducibility: from where was DSSO purchased? For crosslinking, what was the estimated input (i.e. # cells used to isolate mitochondria/estimated protein amount as starting material for XL?) How was the final protein concentration after MeOH/Chloroform precipitation determined?

Reviewer #2

(Remarks to the Author)

The manuscript by Pérez-Pañeda et al. addresses the intriguing and novel topic of the link between the ATP synthase complex (Complex V) and certain enzymes of the TCA cycle. This connection between two central metabolic hubs in mitochondria—the oxidative phosphorylation system and the Krebs cycle—is of significant interest.

It is well established that the absence of a functional oxidative phosphorylation system does not abolish TCA cycle activity. However, the interdependence and direct interactions between components of these two systems have not been systematically and structurally explored. The authors employ crosslinking mass spectrometry to reveal potential interactions, which is a suitable technique for identifying proteins in close spatial proximity. Nevertheless, the use of crosslinking alone cannot definitively demonstrate functional interactions, as spatial proximity does not necessarily equate to direct or functional binding, contrary to some claims made in the manuscript.

The authors also use Blue Native Gel Electrophoresis (BNGE) to show co-migration of proteins. While this approach can suggest potential associations, it is widely accepted in the field that co-migration does not prove physical or functional interaction. To robustly demonstrate true interactions, genetic approaches such as specific ablation or knockdown of one partner are necessary. Although the authors include some genetic mutants, these are not specific to the purported interacting partners, limiting the strength of their conclusions.

The manuscript tends to jump to premature conclusions based on the presented data. The authors rush to infer functional implications, such as suggesting that the observed associations represent a common response to ATP synthase instability. However, the disease models used affect multiple components beyond ATP synthase, including other respiratory complexes, supercomplexes, calcium uptake pathways, solute carriers, and mitochondrial tRNA aminoacylation enzymes. This lack of specificity undermines the attribution of observed effects solely to ATP synthase dysfunction. Without appropriate, specific genetic models, these conclusions remain unsupported.

Furthermore, the manuscript lacks functional assays that could validate the biological relevance of the proposed protein interactions. For example, ATPIF1 is known to modulate ATP synthesis and hydrolysis when bound to Complex V, yet the authors do not provide data demonstrating ATPIF1 binding to Complex V in their system. Given that ATPIF1 also has other mitochondrial functions, such as modulating calcium signaling, the absence of binding data weakens their argument.

Regarding mitochondrial ATP hydrolysis, the authors state that only damaged mitochondria hydrolyze ATP. This is inaccurate; healthy mitochondria can also perform ATP hydrolysis, although ATP synthesis is generally favored under physiological conditions.

On line 385, when the authors mention "mitochondrial stress," it is unclear what specific type of stress they refer to. Clarification is necessary for proper interpretation.

Another significant concern is the manuscript's handling of references and the current state of the art. The authors appear to selectively cite their own work while neglecting important foundational studies by other groups. This bias diminishes the manuscript's scholarly rigor and should be addressed.

Overall, the manuscript is primarily descriptive, summarizing crosslinking data and other observations without sufficient genetic or functional validation to support the authors' claims. The conclusions are overstated relative to the presented evidence.

Reviewer #3

(Remarks to the Author)
Uncovering the link between ATP synthase and the TCA cycle by crosslinking mass spectrometry
Laura Pérez Pañeda, Jelena Misic, Tereza Kadavá, Nils-Göran Larsson, Albert J.R. Heck

The authors present an integrative proteomics analysis of the ATP synthase interactome and complex remodeling comparing wild-type and dysfunctional conditions of heart mitochondria by in-solution crosslinking mass spectrometry (XL-MS), bottom-up quantitative proteomics, and BN-PAGE. XL-MS identifies extensive crosslinks between the F_1 catalytic head of ATP synthase and multiple TCA cycle enzymes in wild-type hearts and shows that these interactions significantly increase in Lrpprc knockout mice. BN-PAGE analysis demonstrates altered assembly states of ATP synthase, including the appearance of distinct F_1 subassemblies and reduced levels of ATP synthase dimers in the Lrpprc knockout hearts. Quantitative proteomics determines protein abundance changes, revealing selective downregulation of mtDNA-encoded subunits such as ATP6 and upregulation of several FO-associated nuclear-encoded subunits, providing support for the structural destabilization inferred from XL-MS and BN-PAGE data. Finally, XL-MS detects increased crosslinking of ATIF1 to the F_1 head specifically in the knockout condition, supporting the conclusion that ATP synthase undergoes regulatory remodelling towards an energy-preserving state under conditions of mitochondrial dysfunction. The combination of XL-MS, quantitative mass spectrometry, and BN-PAGE yields a comprehensive dataset that provides insights into the structural, metabolic, and regulatory adaptations of ATP synthase in cardiac mitochondria.

Overall, I appreciate solving the technological challenge of identifying and quantifying a large number of crosslinks covering the protein complexes of the respiratory chain, the TCA cycle and regulatory proteins by in situ crosslinking of heart mitochondria. Furthermore, the crosslinking data identify the ATP Synthase F1 subcomplex as a novel interphase between the respiratory chain and the TCA cycle besides complex II which further supports the model of multi-enzyme super-complex formation for the efficient channelling of substrates / metabolites in metabolic pathways. However, the structural interpretation of ATP synthase complex remodelling, conformational changes and altered oligomeric states of ATIF1 is solely based on correlating data with previously published observations. The mechanistic/functional interpretation based on these correlative observations is quite speculative and could be a result of an over-interpretation of the data. Thus, I recommend a major revision of the study providing complementary data substantiating the conclusions or the transfer of the manuscript to a more specialized journal.

Major comments:
The manuscript reports substantial changes in ATP synthase interactions and complex formation in the Lrpprc knockout model, including stronger contacts with TCA cycle enzymes and ATIF1. While the XL MS measurements do show these interactions changes, several mechanistic interpretations—such as a functional metabolon (lines 310–329) or pH driven ATIF1 activation (lines 250–257, 371–374) - rely more on inference than on direct analyses in this system. Since these two conclusions are central to how the authors deduce the biological implications, it requires additional data and a clear distinction of what is based on correlation with previously published data, what is a hypothesis, and what is specifically supported by the presented data.

• Line 278–280: The claim about increased ATP5I and ATP5L intralinks is not entirely clear from Figure 5. The authors note later in the paragraph that these subunits also increase in abundance, but this caveat comes after the interpretation, even though it potentially changes how the result should be understood. Figure 5 shows changes in interlinks (Fig. 5A) and ATP synthase crosslinks (Fig. 5B). While intralinks look elevated, the proteomics data also show higher ATP5I/ATP5L levels, so the increase may reflect abundance rather than structural remodeling.

• Line 316-318: "Of note, cross-species reanalyses of XL-MS data, including boar sperm, bovine heart, and human HEK293T mitochondria, confirmed that the F_1 catalytic head of ATP synthase universally forms extensive contacts with multiple TCA enzymes (references 49–51)."

Reference 49: does not mention crosslinks between TCA enzymes and ATP synthase and the only crosslinks described involve outer mitochondrial membrane arrays composed of glycerol kinase like proteins
Reference 50: in this paper crosslinks between Cytochrome c oxidase (COX, Complex IV in OXPHOS) and apoptosis-inducing factor 1 (AIFM1) are shown but ATP-synthase/CV or other TCA cycle enzymes are not mentioned
Reference 51: the paper deals with the establishment of a new method CLASP and chooses two F1-subunits as localization markers, there are no links reported to TCA cycle enzymes

To me it is also unclear how the reanalyses were performed and why the identified interactions were missed by the previous studies.

o The formation of F1 subassemblies is indicated by BN-PAGE and correlates with data from XL-MS and quantitative mass spectrometry, however, the protein composition of the these BN protein bands has not been investigated and is also not fully clarified by the reanalysis of previous profiling data in figure S10. Analysis of the protein composition of these subassemblies and clarification of what these assemblies are composed of, or how they compare to known F_1 intermediates, would possibly allow a more unequivocal interpretation of the data.
o Reference 15: argues that the OXPHOS dysfunction in LRPPRC is based on the deficiency of ATP synthase and describes the two F1 subassemblies, however does not show/discuss the detachment of the F1-head

• Similarly, the shift from higher oligomeric states to the ATIF1 dimer due to a drop in pH explains the lack of "Intralinks" in the N-terminal inhibitory region under conditions of ATP synthase dysfunction but probably could be also a consequence of other interactions / reorganization processes. In the same context, it would be interesting whether these mitochondria preparations also reproduce the phosphorylation pattern - dephosphorylation of S39 in the N-terminal inhibitory region in Lrpprc knockout mice.

Minor Comments
• Line 54: typo …. relies on
• Line 231–238: The connection between increased PPIF–ATPB interactions and higher susceptibility to PTP opening is speculative without either a functional assay or a reference.
• Line 251–256: The description of pH dependent ATIF1 rearrangement and proton gradient collapse in the Lrpprc knockout without any further experiments need to mention that neither matrix pH nor ATIF1 oligomeric state is directly assessed here.
• Figure 1F: in the zoomed in ATP synthase subunits, one of them appears to be mislabelled as ATP68, assuming it should be ATP6
• in general: the manuscript may benefit from a schematic representation of the individual complexes, subunits, domains, enzymes and regulators early on to better understand the frequent references to ATP synthase subunits and structural regions (F_1 head, F_0 membrane domain, peripheral stalk, ATP5I/ATP5L/ATP8, ATPO, ATP5J) as keeping track of the spatial relationships becomes challenging for a non-expert.

Version 1:

Reviewer comments:

Reviewer #1

(Remarks to the Author)
The authors clearly improved their manuscript upon revision by adding previously missing information, clarifying where needed and by addition of new experimental data. They addressed most questions raised by the reviewers, with those not fully addressed indeed being outside the scope of this proteomics/crosslinking mass spectrometry based study in my opinion. I can now recommend their study for publication.

Reviewer #2

(Remarks to the Author)
The revised manuscript by Perez-Paneda et al. includes additional experiments supporting the role of Lrpprc in maintaining ATPase integrity. However, the authors have not incorporated any experiments directly assessing the contribution of TCA cycle enzymes to this process. While they argue that generating mouse models is time-consuming and labor-intensive—which is indeed the case—this dependency could be explored using cell culture systems.

Their claim that ablation of TCA cycle enzymes may broadly disrupt cellular metabolism and produce pleiotropic effects is valid. Nevertheless, the model they employ is not restricted to a single target and may itself introduce multiple effects.

Lrpprc primarily regulates mitochondrial mRNA stability and translation, thereby indirectly supporting the assembly and integrity of oxidative phosphorylation complexes, including ATP synthase through proper expression of mtDNA-encoded subunits such as ATP6 and ATP8. However, its connection to the TCA is only indirect and arises as a secondary consequence of impaired respiratory chain function, rather than direct regulation of TCA enzymes. Therefore, any proposed functional link between Lrpprc, ATP synthase integrity, and the TCA cycle remains insufficiently supported without direct experimental evidence, that are not shown in the manuscript. Notably, no direct measurements of TCA cycle activity are presented. As such, the proposed functional link between ATP synthase and the TCA cycle is not experimentally supported and relies solely on putative interactions.

Furthermore, the manuscript title does not accurately reflect the research presented and may be misleading. The proposed link between ATPase and the TCA cycle is indirect, relying on a protein with multiple roles in mitochondrial function. At present, there is no direct evidence supporting this dependency beyond mass spectrometry data.

Reviewer #3

(Remarks to the Author)
The authors have addressed my comments in quite some detail, provided additional data / reanalysis and rephrased the text accordingly. Still, I believe that some additional functional insights would have strengthened the findings concluded from the crosslinking mass spectrometry data. Overall, I suggest publishing this work in Nature Communications.

Open Access This Peer Review File is licensed under a Creative Commons Attribution 4.0 International License, which permits use, sharing, adaptation, distribution and reproduction in any medium or format, as long as you give appropriate credit to the original author(s) and the source, provide a link to the Creative Commons license, and indicate if changes were made.
In cases where reviewers are anonymous, credit should be given to 'Anonymous Referee' and the source.
The images or other third party material in this Peer Review File are included in the article's Creative Commons license, unless indicated otherwise in a credit line to the material. If material is not included in the article's Creative Commons license and your intended use is not permitted by statutory regulation or exceeds the permitted use, you will need to obtain permission directly from the copyright holder.
To view a copy of this license, visit https://creativecommons.org/licenses/by/4.0/

Original reviewer comments are in black, our responses in blue

REVIEWER COMMENTS

Reviewer #1 (Remarks to the Author):

In their study Paneda et al investigated the structural and functional integration of ATP synthase (Complex V) within the mitochondrial metabolic network. By employing in-solution crosslinking mass spectrometry (XL-MS), quantitative proteomics, and Blue Native PAGE (BN-PAGE), the authors identify a direct physical association between the F1F1 catalytic head of ATP synthase and several TCA cycle enzymes. They further demonstrate that this interaction is strengthened under mitochondrial stress (LRPPRC deficiency) and characterize the regulatory role of the ATPase inhibitory factor 1 (ATIF1).

The manuscript is well written, and conclusive. This study challenges the traditional view that Complex II is the sole structural bridge between the TCA cycle and the OXPHOS system. The discovery of a "metabolon-like" hub centered around ATP synthase is a significant conceptual advance. The observation that these interactions are dynamic and responsive to mitochondrial dysfuncton(LRPPRC, TFAM, and RNase H1 models) adds a layer of physiological relevance. That said, this study is of great interest to the mitochondrial research field and a very nice example of what can be done with XL-MS as a technique. The following points would further strengthen the paper before publication in Nature Communications.

We thank the reviewer for the careful evaluation and support for our work.

1. The authors compare an LRPPRC knockout to its wt analogue and investigate a change in crosslink intensity or counts. At the same time, their quantitative proteomics approach shows regulation of protein in the same direction as the detected crosslinks (i.e. in Figure 2 A vs E). The manuscript would benefit from a more explicit discussion on how they normalized crosslink intensities relative to protein abundance. While they mention using quantitative proteomics as a reference, a more formal "crosslink-to-protein ratio" analysis for key interactions would strengthen the claim that the affinity or conformation has changed, not just the concentration.

We have revised the manuscript to better explain the normalization of crosslink intensities relative to protein abundance. For each crosslinked protein pair, we calculated the maximum absolute protein fold change of the two proteins and plotted this value versus the crosslink fold change. We assumed that the protein with the highest absolute fold change in abundance within a given pair would have the strongest influence on the crosslink intensity and fold change. As shown below in the figure for the reviewer (Fig. R1), the resulting comparison shows no to very weak correlation between protein abundance changes and crosslink fold changes (R^2 0.215). Notably, the protein abundance remains unchanged in most cases while crosslink intensities exhibit a substantial and significant variation. This shows that the observed crosslink changes are mostly not due to altered protein abundances. In Fig. R1 below (red dots), we have highlighted in the protein interactions presented in Fig. 3C of the manuscript. The fold changes of the proteins highlighted in Fig. R1 are included in Supplementary Fig. 5 of the manuscript. Together, these analyses support that the observed crosslink changes reflect genuine changes in interactions rather than changes in protein abundances. Additionally, we would like to clarify that no protein-protein interactions involving proteins with significantly altered abundance were used to draw conclusions unless explicitly stated in the manuscript.

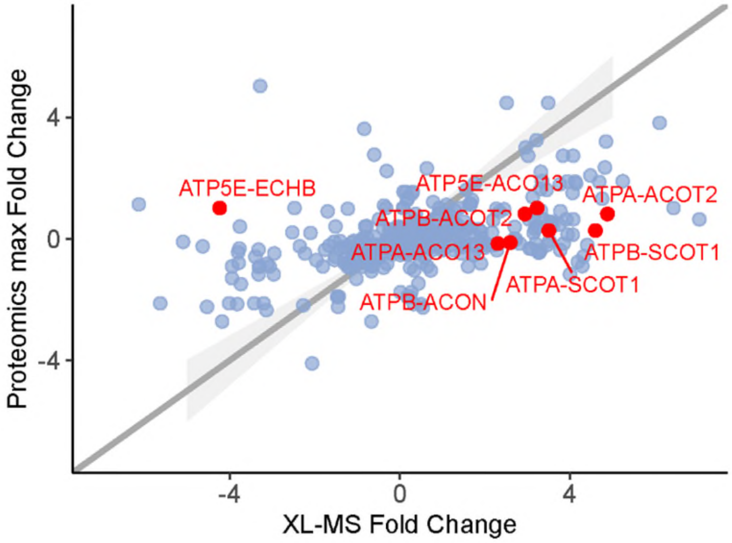

Fig. R1. Changes in crosslink intensities show very weak to no correlation with changes in protein abundances. The protein fold change in intensity was plotted against the crosslink fold change in intensity. The line represents a linear correlation with R^2 equal to 1 with the 20% confidence interval indicated by the gray shaded area. There is at most a very weak correlation between changes in protein abundances and changes in crosslink intensities (R^2 of 0.215).

2. Functional Consequences of the ATP Synthase-TCA Link:The manuscript suggests that this link coordinates ATP synthesis with metabolic flux. While the current study is very strong on structural proteomics its lighter on metabolic flux data. Including (or citing) evidence of altered TCA cycle kinetics or substrate channeling in the LRPPRC model would bridge the gap between "physical link" and "functional coordination." For instance, does the association with MDHM specifically alter the rate of malate oxidation?

We agree with the reviewer that our interaction-based evidence would be even stronger when linked to biological function, and we acknowledge that the current manuscript is not designed to quantify metabolic fluxes (including malate oxidation rates) in the *Lrpprc* knockout hearts. However, there is substantial prior evidence that respiratory chain dysfunction imposes predictable constraints on TCA cycle behavior.

In more detail, when respiratory chain function is impaired (as in the *Lrpprc* knockout hearts, addressed in detail by Mourier et al. [1]), mitochondria have difficulty reoxidizing NADH to NAD^+. As a result, the matrix becomes “more reduced” (high NADH/NAD^+ ratio) [2], which tends to slow down the oxidative steps of the TCA cycle that require NAD^+. Consistent with this principle, Altea-Manzano et al. [3] showed that in respiration-deficient ρ0 cells (cells lacking mtDNA leading to loss of OXPHOS), malate dehydrogenase (MDHM) can run in reverse direction, reducing oxaloacetate to malate instead of oxidizing malate to oxaloacetate, as a redox adaptation that helps regenerate mitochondrial NAD^+ to sustain mitochondrial metabolism. In addition, *in vivo* isotope-tracing studies in patients with clear cell renal cell carcinoma have shown that reduced mitochondrial respiratory chain capacity is associated with decreased oxidative TCA cycle turnover, supporting the broader concept that respiratory chain limitation can suppress oxidative TCA flux [4].

Taken together, these earlier studies provide a framework for interpreting and validating our observations. In *Lrpprc* knockout heart mitochondria, the increased association between ATP synthase (particularly the F_1 domain) and multiple TCA cycle enzymes, including MDHM, is most consistent with a general remodeling of mitochondrial matrix metabolism under respiratory chain stress and redox limitation, rather than a specific change in malate oxidation per se. We therefore speculate these strengthened interactions between the F_1 head and TCA cycle enzymes as potential structural readout of respiratory chain limitation that could help coordinate ATP synthase rate with TCA cycle organization. We recognize that direct flux measurements will be required to test this hypothesis and are beyond the scope of this manuscript.

We incorporated the here mentioned literature in the revised manuscript to better link the observed physical association to expected functional consequences of respiratory chain deficiency caused by *Lrpprc* deletion.

3. Figure 2D vs E: The authors describe that the ATPA-MDHM interaction has the strongest change of #CSM in KO vs wt. This is further confirmed by native PAGE western blot (Figure 2F). However, in the quantitated XL data (Figure 2E) it seems that other interactions highlighted there are regulated at much higher fold change and with a better p value. Do you have any hypothesis why the number of CSMs did not change accordingly even stronger for those candidates? Can you check the "regulation" by CSM counting at least of some of the ATP synthase-OM links as well to compare and get a better estimate of the reliability of the applied MS1-based intensity comparison for the purpose of crosslink quantification. I think this is of special importance as crosslink quantification is done quite less frequently compared to generation of pure qualitative information in the XL-MS field. In conclusion, this reflects a potential easy to apply and effective strategy for others from technical perspective. Further, other findings within this study (i.e. Figure 5) are entirely based on this quantification approach hence its reliability should be clearly shown.

We initially prioritized the ATP synthase F_1-MDHM association because MDHM-derived oxaloacetate has been reported to modulate respiratory-chain flux through feedback inhibition of complex II [5]. In the manuscript, when we refer to the "strongest increases in crosslink intensity" for ATPA-MDHM, we specifically mean the change in crosslink intensity, not the number of CSMs. We agree that other interactions show higher fold changes and/or stronger statistical support and may also be accompanied by larger differences in CSM counts, we have clarified this wording accordingly.

"We focused on the F_1 subunit ATPA and the TCA cycle enzyme MDHM, as they exhibited one of the strongest increases in crosslink intensity among TCA cycle enzymes between the two systems (Fig. 3C)."

To address the relation of CSM counting and intensity-based fold change of crosslinks, we plotted in Fig. R2 all the CSMs detected (and quantified) for the protein interactions highlighted in Fig. 3C. In all cases, the CSMs agree with the comparison provided by the crosslink intensities, and proteins that have higher p-value and fold change than ATPA-MDHM correspond to on/off situations in which the interaction was detected in only one of the conditions. Additionally, the intensity-based quantification enables identifying ATPA-ACO13 as upregulated in the knockout due to differences in crosslink intensity. This observation demonstrates the need to use intensity-based quantification instead of spectral counting (CSMs), because it has been shown that spectral counting provides a poor correlation to protein abundance when few spectral counts are detected [6]. Therefore, intensity-based crosslink quantification mitigates the problem of comparing changes in samples with such a big dynamic range of crosslink abundances.

To further demonstrate the reliability and reproducibility of the crosslink quantification, we evaluated the correlation of the crosslink intensities across biological replicates. Specifically, we calculated the pairwise correlations of log2-normalized intensities. As shown in Fig. R3, all comparisons yielded correlation coefficients with R^2> 0.8 in all cases, indicating strong agreement between replicates. These results demonstrate high reproducibility of crosslink quantification across independent replicates and support the robustness of this quantification strategy.

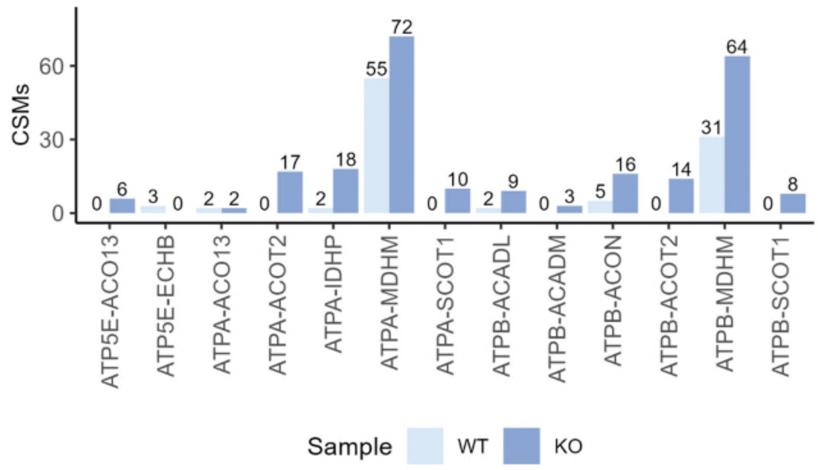

Fig. R2. Crosslink spectral matches (CSMs) detected for all interactions highlighted in Fig. 3C. Data represents the sum of n=3 biological replicates from wild-type (light blue) and *Lrpprc* knockout (dark blue) mice.

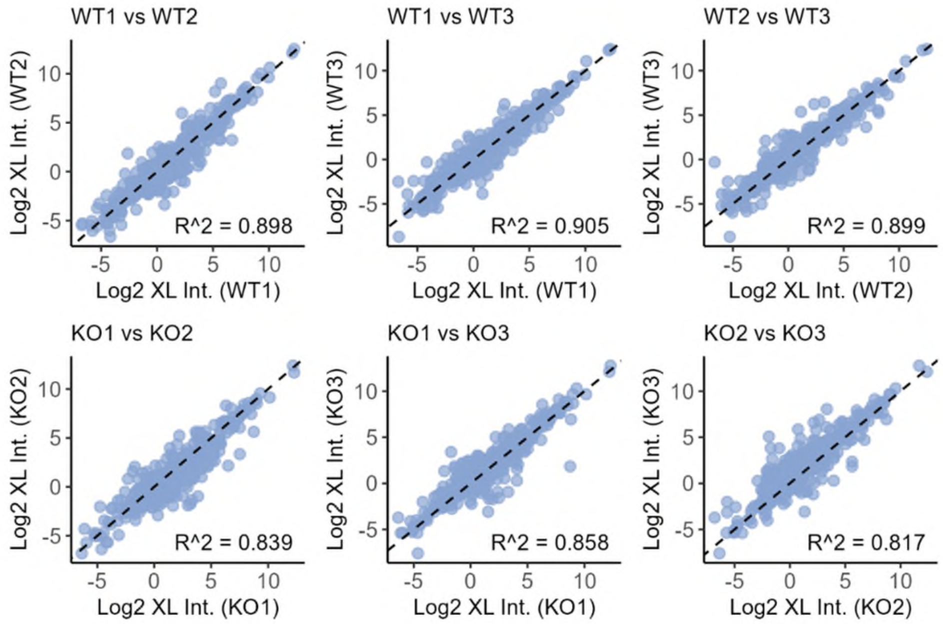

Fig. R3. Crosslink (XL) intensities show high reproducibility across replicates. The normalized log2 protein crosslink intensities were compared across replicates, showing high reproducibility as indicated by their correlation coefficient (R^2).

4. To that end, in the methods part, the authors describe: “Crosslinked peptides were quantified by the addition of the MS1 precursor intensities across all spectral matches. Crosslinks without precursor intensities were excluded for analysis” which makes sense, but it would be important to add more details here to allow reproducibility. For fold change calculations: in how many replicates did the crosslinks had to be quantified for being used for fold changes and volcano plots? Was any normalization or filter, next to the mentioned score threshold of 40 for XlinkX IDs, applied (i.e. a minimal signal/noise)? How was readout of MS1 quantities done, is this reported by XlinkX in PD, or was a custom script/different software used? If so, can you share the code?

We now provide further details on the crosslink quantification and have adjusted the MS data analysis section in the Methods as follows:

“The MS1 intensity information was retrieved from the MaxQuant table “allPeptides.txt”, previously generated in the proteomics search. The crosslink identifications extracted from the XlinkX “PSMs” table were combined with the feature MS1 intensities from the MaxQuant “allPeptides.txt” table, based on the raw file name and the MSMS scan number. Therefore, generating a table containing all the XL-MS information from XlinkX and the MS1 intensities from MaxQuant.”

The normalization and imputation strategies are described in the "Statistical analysis of proteomics and crosslinking data section", now renamed "Quantification of changes in proteins and protein-protein interactions" in Methods.

5. Methods, XL of mitochondria: How was DSSO added (as stock solution at given concentration in DMSO?). Since the quality purity and hence needed optimal molarity differs a lot from vendor to vendor in my experience, for reproducibility: from where was DSSO purchased? For crosslinking, what was the estimated input (i.e. # cells used to isolate mitochondria/estimated protein amount as starting material for XL?) How was the final protein concentration after MeOH/Chloroform precipitation determined?

We have included additional details in the "crosslinking of mouse heart mitochondria" Methods section to clarify the points raised by the reviewer:.

"Intact mouse heart mitochondria (500 µg) purified from three biological replicates of wild-type and Lrpprc knockout mice were treated with 0.5 mM DSSO (CF Plus Chemicals) for 45 min at 15°C, followed by quenching for 30 min at 15°C."

Additionally, we provide two exemplary LC-UV chromatograms acquired during sample fractionation in Fig. R4 below, which shows that both wild-type and knockout samples have similar chromatographic profiles and intensities.

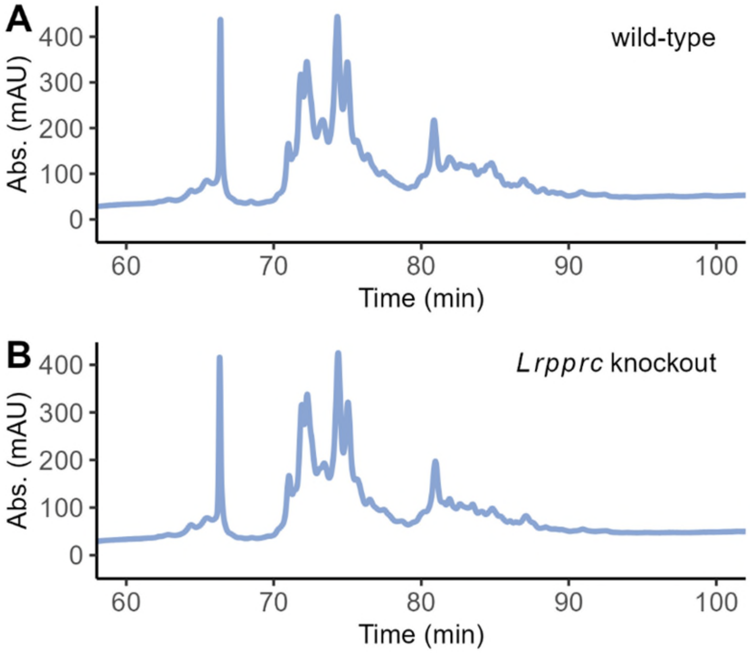

Fig. R4. LC-UV chromatograms of wild-type and *Lrpprc* knockout samples display similar elution profiles. The chromatograms show no difference in intensity or elution profile of the (crosslinked) peptides. The chromatograms were acquired by LC-UV (wavelength = 218 nm) during a 120 min gradient using a strong cation exchange column for sample separation and posterior fractionation.

Reviewer #2 (Remarks to the Author):

The manuscript by Pérez-Pañeda et al. addresses the intriguing and novel topic of the link between the ATP synthase complex (Complex V) and certain enzymes of the TCA cycle. This connection between two central metabolic hubs in mitochondria—the oxidative phosphorylation system and the Krebs cycle—is of significant interest.

It is well established that the absence of a functional oxidative phosphorylation system does not abolish TCA cycle activity. However, the interdependence and direct interactions between components of these two systems have not been systematically and structurally explored. The authors employ crosslinking mass spectrometry to reveal potential interactions, which is a suitable technique for identifying proteins in close

spatial proximity. Nevertheless, the use of crosslinking alone cannot definitively demonstrate functional interactions, as spatial proximity does not necessarily equate to direct or functional binding, contrary to some claims made in the manuscript.

We thank the reviewer for appreciating the novelty and the significance of this study.

We agree that crosslinking mass spectrometry (XL-MS) reports spatial proximity (i.e., residues captured within the crosslinker's distance constraint) and does not necessarily prove that two proteins engage in a stable and/or functionally causal interaction. Nevertheless, XL-MS is widely used to link changes in protein proximity and complex architecture to different functional states [7–9]. Importantly, our conclusions do not only rely on XL-MS but we also present biochemical evidence from BN-PAGE comigration. Furthermore, we have performed new experiments and present complexome profiling proteomics data that quantitate ATP synthase assemblies and subassemblies and reveal systematic interactome remodeling in *Lrpprc* knockout hearts (Fig. 2C, E in the revised manuscript). Moreover, several associations revealed here by XL-MS, most notably ATIF1 binding to ATP synthase, are strongly supported by extensive independent structural, biochemical, *in vivo*, and *in vitro* evidence [10–13]. We would also like to emphasize that we do not make strong causal claims based on these data, rather, we present the observed proximity and assembly changes and discuss their possible implications as hypotheses to guide future mechanistic and metabolic studies.

To further support the structural plausibility of the crosslinks detected by XL-MS (without overinterpreting them as functional interactions), we mapped the crosslinks detected in wild-type heart mitochondria onto structural models of ATP synthase and MDHM (Fig. R5). The mapped crosslinks are distributed across multiple regions on both proteins, consistent with a defined spatial relationship between ATP synthase and MDHM rather than a single isolated contact and showing that the observed crosslinks are compatible with known protein structures.

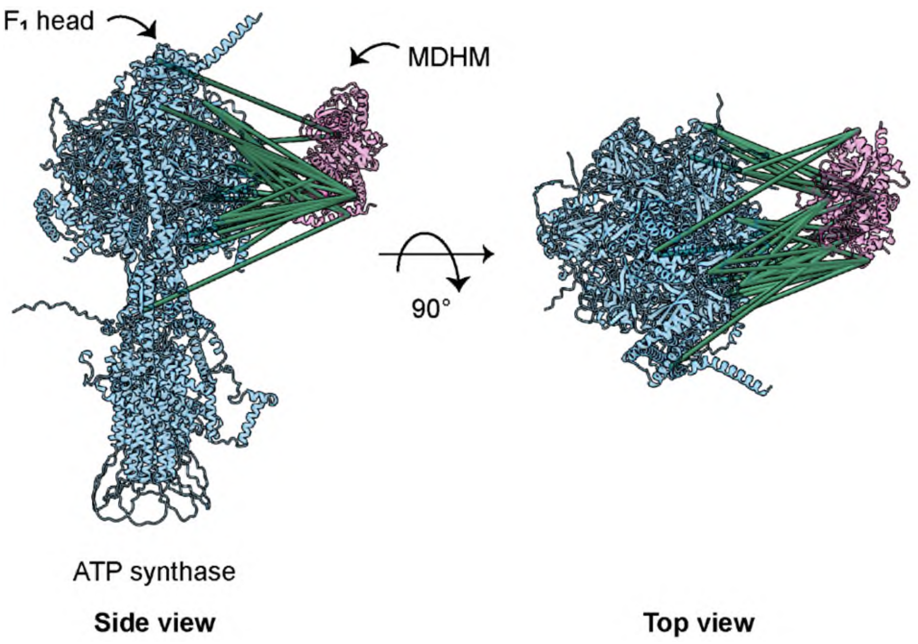

Fig. R5. ATP synthase-MDHM interactions visualized on structural models. The models depict all interlinks detected between the two structures: ATP synthase (blue) and MDHM (pink). The ATP synthase structure was obtained by superposition of the individual components over the Sus scrofa cryo-EM structure (PDB: 6J5K). The MDHM dimer was generated using AlphaFold 2. All crosslinks correspond to the shortest possible restraint with allowed difference of 2 Å.

The authors also use Blue Native Gel Electrophoresis (BNGE) to show co-migration of proteins. While this approach can suggest potential associations, it is widely accepted in the field that co-migration does not

prove physical or functional interaction. To robustly demonstrate true interactions, genetic approaches such as specific ablation or knockdown of one partner are necessary. Although the authors include some genetic mutants, these are not specific to the purported interacting partners, limiting the strength of their conclusions.

The manuscript tends to jump to premature conclusions based on the presented data. The authors rush to infer functional implications, such as suggesting that the observed associations represent a common response to ATP synthase instability. However, the disease models used affect multiple components beyond ATP synthase, including other respiratory complexes, supercomplexes, calcium uptake pathways, solute carriers, and mitochondrial tRNA aminoacylation enzymes. This lack of specificity undermines the attribution of observed effects solely to ATP synthase dysfunction. Without appropriate, specific genetic models, these conclusions remain unsupported.

We agree with the reviewer that BN-PAGE comigration does not necessarily demonstrate direct physical binding or functional causality. However, in the manuscript, we present BN-PAGE as supporting evidence that is consistent with the proximity relationships detected by XL-MS, rather than as proof of a functional interaction. We have also performed new experiments and present complexome profiling proteomics of BN-PAGE gels that complements the XL-MS proximity information and validate that the relevant proteins comigrate in native assemblies and that these assemblies are systematically remodeled in the *Lrpprc* knockout hearts.

We also acknowledge that targeted genetic perturbation of individual putative partners (e.g., ablation of a specific TCA enzyme or ATP synthase subunit) can be valuable for further mechanistic dissection. However, generating and validating additional mouse models with partner-specific perturbations is not trivial. Such studies are costly and may take several years to complete, without any guarantee of success. Furthermore, such perturbations would not provide a clean test of the proposed associations because the function of the TCA cycle and OXPHOS system are tightly linked. Therefore, deleting a single TCA enzyme or ATP synthase subunit would be expected to disrupt mitochondrial metabolism broadly, making it difficult to determine whether any resulting phenotype is due to loss of a specific protein-protein association or a systemic metabolic defect. In addition, many core metabolism genes are essential, and their deletion typically results in embryonic lethality. Further studies may therefore require complex tissue-specific knockout strategies, which will additionally extend the time needed for proper model development. Because of these reasons, we believe that additional partner-specific genetic models, while valuable for future work, are not required to support the main conclusions of the present study and may not directly resolve the issues raised. Instead, we focused on the established heart- and skeletal muscle-specific *Lrpprc* knockout mouse model, for which the bioenergetic consequences of impaired mitochondrial gene expression were characterized in detail previously [1]. It has been demonstrated that the main bioenergetic defect in this model is caused by deficiency of ATP synthase, characterized by loss of fully assembled ATP synthase, accumulation of F_1 subassemblies, and impaired coupling of ATP turnover to proton translocation. In the revised manuscript, we further extend the molecular characterization of this mouse model by experimentally defining the ATP synthase assemblies and subassemblies with proteomics-based complexome profiling. Together, this allows us to place our structural and interactome findings in a well-defined functional context of ATP synthase deficiency and support the use of LRPPRC knockout model to investigate how ATP synthase dysfunction reshapes its protein interaction network.

Finally, we also added a dedicated paragraph in the manuscript outlining the main limitations of the study and clarifying which conclusions are supported by the current data:

"The data presented in this study reveal extensive and intricate interactions between ATP synthase and multiple TCA cycle enzymes in situ and how they are remodeled when ATP synthase function and integrity are compromised. Future functional studies will be required to define the metabolic consequences of these interactions. In addition, the quantitative XL-MS workflow introduced in this study provides new opportunities for the crosslinking community. Further refinement of the data filtering procedures and implementation of more advanced normalization strategies may enable recovery of additional protein interactions, addressing a persistent challenge in XL-MS."

Furthermore, the manuscript lacks functional assays that could validate the biological relevance of the proposed protein interactions. For example, ATPIF1 is known to modulate ATP synthesis and hydrolysis when bound to Complex V, yet the authors do not provide data demonstrating ATPIF1 binding to Complex V in their system. Given that ATPIF1 also has other mitochondrial functions, such as modulating calcium signaling, the absence of binding data weakens their argument.

We thank the reviewer for this important comment. Several lines of experimental evidence address the issues raised:

1. First, we have included *in-gel* ATPase activity assays (Fig. 4A) showing that ATP hydrolysis is associated not only with ATP synthase monomers but also with ATPase-active F_1 assemblies in *Lrpprc* knockout heart mitochondria. Moreover, in previously published work in the same *Lrpprc* knockout model, we performed similar native-gel ATPase assays in the presence and absence of oligomycin, an inhibitor of the F_O domain that suppresses ATP hydrolysis by intact F_1F_O ATP synthase [14,15] and observed presence of oligomycin-insensitive ATPase-active F_1 (Fig. 3A–C and 3E in ref [1]). The detached F_1 species can thus hydrolyze ATP independently of the F_O domain.
2. Second, to directly dissect which ATP synthase assemblies/subassemblies ATIF1 comigrates with in the *Lrpprc* knockout samples, we performed new complexome profiling experiments that are included in the revised manuscript (new Fig. 5C). We plotted the migration profiles of ATP synthase F_1, F_O and peripheral stalk subunits and ATIF1 (each normalized to its maximum intensity per lane; new Fig. 5C). In wild-type heart mitochondria, ATIF1 is detected both as a free, low-molecular-weight species and in higher-molecular-weight fractions comigrating with assembled ATP synthase, including monomers, dimers and oligomers. This is consistent with the cryo-EM structure of the mammalian ATP synthase tetramer, in which ATIF1 dimers bridge two ATP synthase dimers within the tetrameric assembly, supporting a role for ATIF1 in higher-order ATP synthase organization in addition to its inhibitory binding to the F_1 catalytic domain [10]. In contrast, in *Lrpprc* knockout hearts, the free ATIF1 pool is notably reduced, as is the fraction comigrating with oligomeric ATP synthase, whereas ATIF1 is enriched in fractions corresponding to F_1 detached species. For a detailed description of the ATP synthase assemblies identified in LRPPRC knockout hearts, please see the response to reviewer 3.
3. Third, our XL-MS data show a clear redistribution of ATIF1 inter- and intralinks across the ATIF1 sequence in *Lrpprc* knockout hearts (Fig. 5E and Supplementary Fig. 9). Notably, interlinks between ATIF1 and F_1 head subunits were specifically detected within the N-terminal inhibitory region of ATIF1 in the *Lrpprc* knockout hearts, suggesting that ATIF1 is positioned to inhibit ATP hydrolysis by the detached F_1 head.

Together, these results support that loss of LRPPRC destabilizes ATP synthase, leading to the accumulation of the detached ATPase-active F_1 head and a shift of ATIF1 toward this species. ATP hydrolysis by detached F_1 head can still be detected in *in-gel* activity assays because ATIF1-mediated inhibition of F_1 is not

necessarily complete, and the substantial accumulation of detached F_1 likely exceeds available ATIF1, leaving some pool of detached F_1 uninhibited or only partially inhibited.

We agree that ATIF1 may have additional mitochondrial roles (including links to Ca^{2+} signaling), and we have revised the manuscript to include relevant references.

Regarding mitochondrial ATP hydrolysis, the authors state that only damaged mitochondria hydrolyze ATP. This is inaccurate; healthy mitochondria can also perform ATP hydrolysis, although ATP synthesis is generally favored under physiological conditions.

The reviewer is correct in pointing this out and we acknowledge that ATP hydrolysis by ATP synthase is not exclusive to damaged mitochondria. Because ATP synthase is reversible, it can "run backwards" and hydrolyze ATP when the proton-motive force (Δp) is low or collapses. In intact mitochondria, this can happen during transient drops in Δp, limited substrate or oxygen availability, ischemia-like conditions, or experimentally when respiration is constrained. We have revised the text in the manuscript to clarify this issue.

On line 385, when the authors mention "mitochondrial stress," it is unclear what specific type of stress they refer to. Clarification is necessary for proper interpretation.

We agree that the term "mitochondrial stress" is unspecific and we have therefore revised the text in the manuscript to clarify that we refer to respiratory chain dysfunction resulting from altered mitochondrial gene expression.

Another significant concern is the manuscript's handling of references and the current state of the art. The authors appear to selectively cite their own work while neglecting important foundational studies by other groups. This bias diminishes the manuscript's scholarly rigor and should be addressed

We appreciate the reviewer's point and aim to take away any potential bias. However, the heart- and skeletal muscle-specific *Lrpprc* knockout mouse was developed in our laboratory and has been used in multiple studies to address diverse aspects of mitochondrial gene expression defects and their biological consequences [1,16–18]. For this reason, several citations in the manuscript refer to prior work from our own group describing the model and its established phenotypes. In line with the reviewer's request, we have now included more references from other groups studying the role of LRPPRC and ATIF1 in mitochondria.

Overall, the manuscript is primarily descriptive, summarizing crosslinking data and other observations without sufficient genetic or functional validation to support the authors' claims. The conclusions are overstated relative to the presented evidence.

We agree that the present work is descriptive, and that XL-MS (together with BN-PAGE and our new complexome profiling data) does not on its own establish functional causality. However, the strength of this study is that it delivers the first integrated *in situ* map of ATP synthase-TCA cycle proximity relationships and shows that this network is remodeled when mitochondrial gene expression is impaired. By combining interaction constraints (XL-MS) with independent native-assembly readouts (BN-PAGE and complexome profiling), we anchor the organizational changes in a clearly defined structural remodeling of ATP synthase caused by *Lrpprc* depletion in the heart. We revised the manuscript to avoid overstated claims and to clearly distinguish proximity or association from causality. We also outlined the study's limitations as an independent section in the manuscript. Overall, we believe that the findings we present here will be of broad interest to the metabolism field and stimulated future targeted mechanistic and functional follow-up studies.

Reviewer #3 (Remarks to the Author):
Uncovering the link between ATP synthase and the TCA cycle by crosslinking mass spectrometry Laura Pérez Pañeda, Jelena Misic, Tereza Kadavá, Nils-Göran Larsson, Albert J.R. Heck
The authors present an integrative proteomics analysis of the ATP synthase interactome and complex remodeling comparing wild-type and dysfunctional conditions of heart mitochondria by insolution crosslinking mass spectrometry (XLMS), bottomup quantitative proteomics, and BNPAGE. XLMS identifies extensive crosslinks between the F_1 catalytic head of ATP synthase and multiple TCA cycle enzymes in wildtype hearts and shows that these interactions significantly increase in ockout mice. BNPAGE analysis demonstrates altered assembly states of ATP synthase, including the appearance of distinct F_1 subassemblies and reduced levels of ATP synthase dimers in the Lrpprc knockout hearts. Quantitative proteomics determines protein abundance changes, revealing selective downregulation of mtDNAencoded subunits such as ATP6 and upregulation of several FOassociated nuclear-encoded subunits, providing support for the structural destabilization inferred from XLMS and BN-PAGE data. Finally, XLMS detects increased crosslinking of ATIF1 to the F_1 head specifically in the knockout condition, supporting the conclusion that ATP synthase undergoes regulatory remodelling towards an energy preserving state under conditions of mitochondrial dysfunction. The combination of XLMS, quantitative mass spectrometry, and BNPAGE yields a comprehensive dataset that provides insights into the structural, metabolic, and regulatory adaptations of ATP synthase in cardiac mitochondria.
Overall, I appreciate solving the technological challenge of identifying and quantifying a large number of crosslinks covering the protein complexes of the respiratory chain, the TCA cycle and regulatory proteins by in situ crosslinking of heart mitochondria. Furthermore, the crosslinking data identify the ATP Synthase F1 subcomplex as a novel interphase between the respiratory chain and the TCA cycle besides complex II which further supports the model of multi-enzyme super-complex formation for the efficient channelling of substrates / metabolites in metabolic pathways. However, the structural interpretation of ATP synthase complex remodelling, conformational changes and altered oligomeric states of ATIF1 is solely based on correlating data with previously published observations. The mechanistic/functional interpretation based on these correlative observations is quite speculative and could be a result of an over-interpretation of the data. Thus, I recommend a major revision of the study providing complementary data substantiating the conclusions or the transfer of the manuscript to a more specialized journal.
We thank the reviewer for the careful evaluation of our manuscript and for recognizing both the technical advance in quantitative *in situ* XL-MS and the potential biological relevance of the findings. We agree that some of our original interpretations relied on correlating the XL-MS results with prior observations and we have therefore strengthened the revised manuscript with new experimental data and revised the text accordingly. We have performed proteomics-based complexome profiling of wild-type and *Lrpprc* knockout heart mitochondria under mild solubilization conditions. These new data (now incorporated into Fig. 2, 5) quantitatively define the distribution of ATP synthase assemblies and subassemblies. For details, please see additional comments below as well as the response to the reviewer 2.
Major comments:
The manuscript reports substantial changes in ATP synthase interactions and complex formation in the Lrpprc knockout model, including stronger contacts with TCA cycle enzymes and ATIF1. While the XL MS measurements do show these interactions changes, several mechanistic interpretations—such as a functional metabolon (lines 310–329) or pH driven ATIF1 activation (lines 250–257, 371–374) - rely more on inference than on direct analyses in this system. Since these two conclusions are central to how the authors deduce the biological implications, it requires additional data and a clear distinction of what is

based on correlation with previously published data, what is a hypothesis, and what is specifically supported by the presented data.

We have revised the text to clarify that we did not measure matrix pH in the current work and that we refer to the pH-dependent ATIF1 regulation only as an established *in vitro* mechanism:

"ATIF1 was shown to undergo pH-dependent oligomeric rearrangements in vitro, such that at physiological pH values above 6.7 it predominantly adopts an inactive tetrameric/oligomeric state composed of two or more dimers linked via the N-terminal inhibitory region (Fig. 5D). Consistent with this in vitro model …"

• Line 278–280: The claim about increased ATP5I and ATP5L intralinks is not entirely clear from Figure 5. The authors note later in the paragraph that these subunits also increase in abundance, but this caveat comes after the interpretation, even though it potentially changes how the result should be understood. Figure 5 shows changes in interlinks (Fig. 5A) and ATP synthase crosslinks (Fig. 5B). While intralinks look elevated, the proteomics data also show higher ATP5I/ATP5L levels, so the increase may reflect abundance rather than structural remodeling.

We agree with the reviewer that it could be made clearer that these crosslinks correspond to proteins with significantly changed protein abundances. In Fig. 6B, where these crosslinks are shown, we mentioned that the abundances in the bottom-up proteomics experiment were changed. However, we have now revised the text to emphasize this caveat before offering any possible structural interpretation of the crosslinking data.

"Importantly, the ATP5I and ATP5L were also increased in abundance according to our bottom-up proteomics data (Fig. 2A), indicating that the elevated ATP5I/ATP5L intralinks could partly reflect higher protein levels rather than structural remodeling per se. With this caveat, the elevated intralinks of the F_O membrane domain likely may reflect strengthened interactions between the ATP5I and ATP5L subunits of adjacent ATP synthase monomers, possibly acting as a compensatory mechanism to counteract dimer instability (Fig. 2D, 3D). "

• Line 316-318: "Of note, cross-species reanalyses of XL-MS data, including boar sperm, bovine heart, and human HEK293T mitochondria, confirmed that the F_1 catalytic head of ATP synthase universally forms extensive contacts with multiple TCA enzymes (references 49–51)."

Reference 49: does not mention crosslinks between TCA enzymes and ATP synthase and the only crosslinks described involve outer mitochondrial membrane arrays composed of glycerol kinase like proteins
Reference 50: in this paper crosslinks between Cytochrome c oxidase (COX, Complex IV in OXPHOS) and apoptosis-inducing factor 1 (AIFM1) are shown but ATP-synthase/CV or other TCA cycle enzymes are not mentioned

Reference 51: the paper deals with the establishment of a new method CLASP and chooses two F1-subunits as localization markers, there are no links reported to TCA cycle enzymes
To me it is also unclear how the reanalyses were performed and why the identified interactions were missed by the previous studies.

It is correct that the mentioned publications do not provide an overview of the ATP synthase-TCA cycle interactions because these manuscripts had a different focus. However, the information is available in the publicly deposited datasets and can be accessed through the PRIDE repository with identifiers PXD025562 (Leung, ref. 49), PXD025102 (Hevler, ref. 50), PXD032132 and PXD046382 (Zhu, ref. 51). In the current manuscript, we reanalyzed these datasets and consistently found interactions between the ATP synthase and TCA cycle enzymes as illustrated in Fig. R6 below. The human and bovine datasets show a big overlap in protein interactions that were detected in the mouse dataset. In the boar dataset from Leung et al., interactions between ECHB and ATP synthase subunits are detected. As expected, the depth of the

detected interactions is lower because of the high complexity of the sample (sperm cells) and the lack of mitochondrial enrichment before crosslinking. Due to the low overlap of this dataset with ours, we decided to remove this reference and adjust the manuscript accordingly.

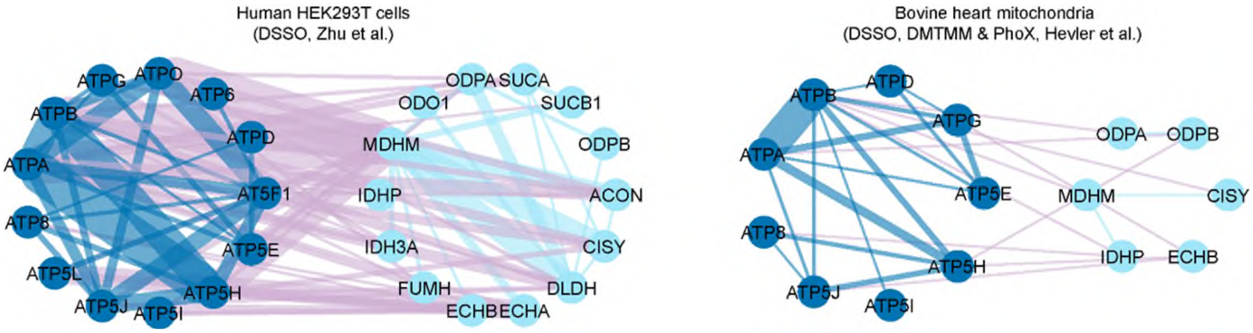

Fig, R6. ATP synthase (complex V) interacts with TCA cycle enzymes in human and bovine mitochondria. Network plot displaying the interactions between the ATP synthase and the TCA cycle enzymes in human HEK293T cells crosslinked with DSSO and bovine heart mitochondria crosslinked in different experiments with DSSO, DMTMM and PhoX. Data represents the sum of n=3 replicates from wild-type mitochondria. To obtain these plots, we reanalyzed data from the PRIDE repository with identifiers PXD025562 and PXD032132, respectively.

• The formation of F1 subassemblies is indicated by BN-PAGE and correlates with data from XL-MS and quantitative mass spectrometry, however, the protein composition of the these BN protein bands has not been investigated and is also not fully clarified by the reanalysis of previous profiling data in figure S10. Analysis of the protein composition of these subassemblies and clarification of what these assemblies are composed of, or how they compare to known F_1 intermediates, would possibly allow a more unequivocal interpretation of the data.

• Reference 15: argues that the OXPHOS dysfunction in LRPPRC is based on the deficiency of ATP synthase and describes the two F1 subassemblies, however does not show/discuss the detachment of the F1-head

We have performed new experiments with complexome profiling proteomics for detailed assessment of the protein composition of ATP synthase assemblies and subassemblies found on PN-PAGE of mitochondria from *Lrpprc* knockout hearts. These new results are incorporated into Fig. 2 and 5 of the manuscript and the corresponding experimental procedures have been added to the Methods section.

In brief, we plotted the migration profiles for the three structural domains of ATP synthase (F_1, F_O and peripheral stalk) calculated as the sum of protein intensities per slice normalized to the maximum protein intensity per lane (new Fig. 2C). In wild-type mitochondria, subunits from all three modules co-migrated predominantly in slices corresponding to monomeric, dimeric, and oligomeric ATP synthase assemblies. In contrast, *Lrpprc* knockout heart mitochondria showed a marked reduction of the dimeric/oligomeric ATP synthase, while the monomeric complex was detected. In addition, we observed accumulation of ATP synthase species composed exclusively of F_1 subunits in two regions of the lane (slices 15–16, corresponding to ~325–390 kDa, and a broad distribution around slices 4–6 corresponding to lower molecular weights), with no comigration of F_O or peripheral stalk components (new Fig. 2E). The selective enrichment of F_1 subunits together with the absence of F_O and stalk subunits supports the notion that these species represent detached F_1 assemblies or even subassemblies. Furthermore, F_O subunits exhibited a distinct low-molecular-weight peak in the *Lrpprc* knockout hearts consistent with fragmentation of the complex. The new results are in line with the functional assays of ATPase activity in

Fig. 4A and the previously published literature [1]. Additional details on the ATPase-active and detached F_1 head are available in the response to reviewer 2.

• Similarly, the shift from higher oligomeric states to the ATIF1 dimer due to a drop in pH explains the lack of "Intralinks" in the N-terminal inhibitory region under conditions of ATP synthase dysfunction but probably could be also a consequence of other interactions / reorganization processes. In the same context, it would be interesting whether these mitochondria preparations also reproduce the phosphorylation pattern - dephosphorylation of S39 in the N-terminal inhibitory region in Lrpprc knockout mice.

We agree with the reviewer that the relationship between ATIF1 interaction rearrangements and its phosphorylation status, including the reported dephosphorylation of S39 in the N-terminal inhibitory region in *Lrpprc* knockout mice, is an interesting question. However, this aspect was not examined in the current study and would require dedicated follow-up analyses beyond the scope of the present manuscript.

Minor Comments

• Line 54: typo …. relies on

Corrected in the manuscript.

• Line 231–238: The connection between increased PPIF–ATPB interactions and higher susceptibility to PTP opening is speculative without either a functional assay or a reference.

To our knowledge, direct binding of PPIF to ATPB has not been reported previously. We therefore agree that a direct connection between the increased PPIF-ATPB interactions observed here and increased susceptibility to PTP opening would be too speculative without functional validation. Because we nevertheless consider this interaction change potentially relevant to the field, we revised the text to make the interpretation more cautious and added an appropriate reference:

"Importantly, PPIF-ATPB interactions were increased in Lrpprc knockout mitochondria, consistent with stress-induced remodeling of the F_1 head and an enhanced susceptibility to permeability transition pore (PTP) activation, previously reported due to PPIF binding to peripheral stalk subunits. "

• Line 251–256: The description of pH dependent ATIF1 rearrangement and proton gradient collapse in the Lrpprc knockout without any further experiments need to mention that neither matrix pH nor ATIF1 oligomeric state is directly assessed here.

We adjusted this, see response above.

• Figure 1F: in the zoomed in ATP synthase subunits, one of them appears to be mislabelled as ATP68, assuming it should be ATP6

It is correctly labeled; ATP68 corresponds to the ATP synthase Fo complex subunit j.

• in general: the manuscript may benefit from a schematic representation of the individual complexes, subunits, domains, enzymes and regulators early on to better understand the frequent references to ATP synthase subunits and structural regions (F_1 head, F_0 membrane domain, peripheral stalk, ATP5I/ATP5L/ATP8, ATPO, ATP5J) as keeping track of the spatial relationships becomes challenging for a non-expert.

Thank you for the suggestion. We agree that the manuscript would benefit from a visual representation of all structural components of ATP synthase and we therefore made a new illustration and included it in the new Fig. 2D.

References used in Rebuttal

1. Mourier, A., Ruzzenente, B., Brandt, T., Kühlbrandt, W. & Larsson, N.-G. Loss of LRPPRC causes ATP synthase deficiency. *Hum. Mol. Genet.* 23, 2580–2592 (2014).
2. Chinopoulos, C. Acute sources of mitochondrial NAD+ during respiratory chain dysfunction. *Exp. Neurol.* 327, 113218 (2020).
3. Altea-Manzano, P. *et al.* Reversal of mitochondrial malate dehydrogenase 2 enables anaplerosis via redox rescue in respiration-deficient cells. *Mol. Cell* 82, 4537-4547.e7 (2022).
4. Bezwada, D. *et al.* Mitochondrial complex I promotes kidney cancer metastasis. *Nature* 633, 923–931 (2024).
5. Molinié, T. *et al.* MDH2 produced OAA is a metabolic switch rewiring the fuelling of respiratory chain and TCA cycle. *Biochim. Biophys. Acta BBA - Bioenerg.* 1863, 148532 (2022).
6. Old, W. M. *et al.* Comparison of Label-free Methods for Quantifying Human Proteins by Shotgun Proteomics*S. *Mol. Cell. Proteomics* 4, 1487–1502 (2005).
7. Lee, B.-G. *et al.* Cryo-EM structures of holo condensin reveal a subunit flip-flop mechanism. *Nat. Struct. Mol. Biol.* 27, 743–751 (2020).
8. Gonzalez-Lozano, M. A. *et al.* Stitching the synapse: Cross-linking mass spectrometry into resolving synaptic protein interactions. *Sci. Adv.* 6, eaax5783 (2020).
9. Zhu, Z., Zhong, X., Wang, B., Lu, H. & Li, L. Probing Protein Structural Changes in Alzheimer's Disease via Quantitative Cross-linking Mass Spectrometry. *Anal. Chem.* 96, 7506–7515 (2024).
10. Gu, J. *et al.* Cryo-EM structure of the mammalian ATP synthase tetramer bound with inhibitory protein IF1. *Science* 364, 1068–1075 (2019).
11. Cabezón, E., Montgomery, M. G., Leslie, A. G. W. & Walker, J. E. The structure of bovine F1-ATPase in complex with its regulatory protein IF1. *Nat. Struct. Mol. Biol.* 10, 744–750 (2003).
12. Cabezon, E., Butler, P. J. G., Runswick, M. J. & Walker, J. E. Modulation of the Oligomerization State of the Bovine F1-ATPase Inhibitor Protein, IF1, by pH*. *J. Biol. Chem.* 275, 25460–25464 (2000).
13. Di Pancrazio, F. *et al.* In vitro and in vivo studies of F0F1ATP synthase regulation by inhibitor protein IF1 in goat heart. *Biochim. Biophys. Acta BBA - Bioenerg.* 1659, 52–62 (2004).
14. Symersky, J., Osowski, D., Walters, D. E. & Mueller, D. M. Oligomycin frames a common drug-binding site in the ATP synthase. *Proc. Natl. Acad. Sci.* 109, 13961–13965 (2012).
15. Pagliarani, A., Nesci, S. & Ventrella, V. Modifiers of the oligomycin sensitivity of the mitochondrial F1F0-ATPase. *Mitochondrion* 13, 312–319 (2013).
16. Ruzzenente, B. *et al.* LRPPRC is necessary for polyadenylation and coordination of translation of mitochondrial mRNAs. *EMBO J.* 31, 443–456 (2012).
17. Rubalcava-Gracia, D. *et al.* LRPPRC and SLIRP synergize to maintain sufficient and orderly mammalian mitochondrial translation. *Nucleic Acids Res.* 52, 11266–11282 (2024).
18. Kühl, I. *et al.* Transcriptomic and proteomic landscape of mitochondrial dysfunction reveals secondary coenzyme Q deficiency in mammals. *eLife* 6, e30952 (2017).

Original reviewer comments are in black, our responses in blue

REVIEWER COMMENTS
Reviewer #1 (Remarks to the Author):

The authors clearly improved their manuscript upon revision by adding previously missing information, clarifying where needed and by addition of new experimental data. They addressed most questions raised by the reviewers, with those not fully addressed indeed being outside the scope of this proteomics/crosslinking mass spectrometry based study in my opinion. I can now recommend their study for publication.

We thank the reviewer for the feedback provided, which helped to improve the quality of our manuscript.

Reviewer #2 (Remarks to the Author):

The revised manuscript by Perez-Paneda et al. includes additional experiments supporting the role of Lrpprc in maintaining ATPase integrity. However, the authors have not incorporated any experiments directly assessing the contribution of TCA cycle enzymes to this process. While they argue that generating mouse models is time-consuming and labor-intensive—which is indeed the case—this dependency could be explored using cell culture systems.

Their claim that ablation of TCA cycle enzymes may broadly disrupt cellular metabolism and produce pleiotropic effects is valid. Nevertheless, the model they employ is not restricted to a single target and may itself introduce multiple effects.

Lrpprc primarily regulates mitochondrial mRNA stability and translation, thereby indirectly supporting the assembly and integrity of oxidative phosphorylation complexes, including ATP synthase through proper expression of mtDNA-encoded subunits such as ATP6 and ATP8. However, its connection to the TCA is only indirect and arises as a secondary consequence of impaired respiratory chain function, rather than direct regulation of TCA enzymes. Therefore, any proposed functional link between Lrpprc, ATP synthase integrity, and the TCA cycle remains insufficiently supported without direct experimental evidence, that are not shown in the manuscript. Notably, no direct measurements of TCA cycle activity are presented. As such, the proposed functional link between ATP synthase and the TCA cycle is not experimentally supported and relies solely on putative interactions.

Furthermore, the manuscript title does not accurately reflect the research presented and may be misleading. The proposed link between ATPase and the TCA cycle is indirect, relying on a protein with multiple roles in mitochondrial function. At present, there is no direct evidence supporting this dependency beyond mass spectrometry data.

The reviewer raises a valid point that LRPPRC deletion primarily impairs mitochondrial gene expression and only secondarily induces ATP synthase dysfunction and instability. Throughout the text, we now clarified this. Please see the examples below.

1. In the abstract: “Together, our findings show that impaired mitochondrial gene expression leads to **secondary** ATP synthase remodeling and reshaping of its interaction landscape, revealing how mitochondria may adapt to bioenergetic stress”.
2. In the results section: “These findings suggest that the enhanced association between ATP synthase and MDHM, used here as a representative TCA cycle enzyme likely reflects a broader response to ATP synthase dysfunction and instability, which arises **secondary** to altered mitochondrial gene expression”.
3. In discussion paragraph 5: “When mitochondrial gene expression is disrupted, as in the heart- and skeletal-muscle-specific Lrpprc knockout model used here, the ATP synthase undergoes a profound **secondary** structural remodeling, as dissected by complexome profiling”.

We would also like to emphasize that enriched crosslinks between ATP synthase and TCA cycle proteins were observed not only upon LRPPRC deletion, but also under wild-type conditions. This suggests that a spatial link between these two systems already exists under basal physiological conditions and is enhanced upon secondary ATP synthase remodeling caused by LRPPRC deletion.

We agree with the reviewer that our data do not provide direct evidence for functional consequences of these interactions. Rather, our findings identify a spatial link between ATP synthase and TCA cycle, both under physiological conditions and in the context of mitochondrial dysfunction caused by impaired mitochondrial gene expression. We now explicitly write that the observed interaction reflects a **spatial link**, rather than a functional link. At the same time, we discuss the possibility that this spatial association may have some functional implications, which will require further experimental validation.

In line with this, we have modified the title from “Uncovering the link between ATP synthase and TCA cycle by crosslinking mass spectrometry” to “Investigating the relationship between ATP synthase and the TCA cycle by cross linking mass spectrometry” to emphasize that our findings are based on XL-MS interaction data.

Reviewer #3 (Remarks to the Author):

The authors have addressed my comments in quite some detail, provided additional data / reanalysis and rephrased the text accordingly. Still, I believe that some additional functional insights would have strengthened the findings concluded from the crosslinking mass spectrometry data. Overall, I suggest publishing this work in Nature Communications.

We thank the reviewer for supporting the publication of our manuscript.
